# Recent Advances in Integrating Graphene into Polymeric Nanocomposite Hydrogels for Biomedical Applications

**DOI:** 10.1002/mabi.202500553

**Published:** 2026-02-13

**Authors:** Abrar Hussain, Irum Batool, Khurram Shahzad, Syed Kumail Hussain Naqvi, Shahzaib Akhter, Ujala Zafar, Khaled Chawraba, Sang Hyun Park

**Affiliations:** ^1^ Advanced Radiation Technology Institute (ARTI) Korea Atomic Energy Research Institute (KAERI) Jeongeup Republic of Korea; ^2^ Radiation Science University of Science and Technology (UST) Daejeon Republic of Korea; ^3^ School of Chemical Engineering and Technology Tianjin University Tianjin China; ^4^ Graduate School of Integrated Energy‐AI Jeonbuk National University Jeonju Republic of Korea; ^5^ Institute of Chemistry University of Sargodha Sargodha Pakistan; ^6^ School of Advanced Materials Science and Engineering Sungkyunkwan University Suwon‐si Republic of Korea; ^7^ Department of Civil and Environmental Engineering The Hong Kong Polytechnic University Hung Hom Kowloon China; ^8^ CNRS, IS2M UMR 7361 University of Haute‐Alsace Mulhouse France

**Keywords:** biomaterials, biomedical applications, graphene‐based hydrogels, machine learning, polymeric nanocomposites

## Abstract

Hydrogels are widely used in biomedical applications because they are biocompatible, respond to external stimuli, and closely mimic the mechanical properties of natural tissues. However, conventional hydrogels often exhibit poor electrical conductivity, low mechanical strength, and limited functionality, hindering their use in advanced biomedical platforms. These challenges can be addressed by integrating 2D graphene and its derivatives into polymer‐based nanocomposite hydrogels (CHGs). Graphene‐enhanced CHGs offer superior mechanical strength, electrical conductivity, thermal stability, and optical properties, making them ideal for advanced biomedical applications. This review provides a comprehensive analysis of graphene's structural and functional properties and its incorporation into hydrogel matrices for CHG synthesis. It explores recent advancements in graphene‐based CHGs for drug delivery, tissue engineering, and photothermal therapy (PTT), highlighting their enhanced performance. Additionally, it examines the emerging role of machine learning (ML) in optimizing CHG properties, such as predictive modeling for wearable sensors in biomedical contexts. By bridging materials science and computational intelligence, this review outlines a roadmap for designing next‐generation smart hydrogels, emphasizing their transformative potential in addressing complex healthcare challenges.

## Graphical abstract

This review explores recent progress in graphene‐based polymeric nanocomposite hydrogels and how they enhance mechanical strength, electrical conductivity, and biocompatibility. It outlines key fabrication strategies and highlights applications in drug delivery, tissue engineering, and photothermal therapy, while also discussing the emerging role of machine learning in designing intelligent hydrogels for future biomedical challenges.

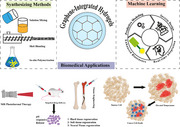

## 1 Introduction

Hydrogels are hydrophilic, 3D cross‐linked molecular networks, typically composed of natural or synthetic polymers and stabilized by covalent or non‐covalent interactions [1]. Non‐covalent interactions include physical entanglements, hydrogen bonding, hydrophobic interactions, supramolecular forces, electrostatic attractions, and coordination bonds [2]. These networks exhibit unique physicochemical properties, such as high water content, wettability, biocompatibility, stretchability, self‐healing, and responsiveness to environmental stimuli like pH and temperature [3, 4, 5]. Their tissue‐like elasticity and compatibility with biological systems [6, 7] make hydrogels ideal for biomedical applications, including drug delivery, wound healing, antimicrobial therapies, and tissue engineering [8, 9].

Despite their advantages, conventional hydrogels have limitations, including insufficient mechanical strength, limited strain tolerance, low thermal and electrical conductivity, and restricted biochemical functionality [10, 11, 12, 13, 14]. These drawbacks hinder their use in advanced biomedical platforms requiring mechanical robustness, electrical conductivity, and multifunctionality. Polymer nanocomposites (NCs) provide a promising solution by incorporating nanoscale fillers, such as graphene, into a polymer matrix to enhance mechanical, thermal, electrical, and biological properties [15]. In hydrogels, biocompatible polymers like polyvinyl alcohol (PVA), chitosan (CS), polyethylene glycol (PEG), and gelatin (Gel) are preferred for their hydrophilicity and tunable crosslinking [16]. The choice of polymer depends on biodegradability, mechanical compatibility, tissue adhesion, and responsiveness to stimuli such as pH, temperature, or light [17]. Effective nanofiller integration requires strong polymer–filler interactions, achieved through methods like in situ polymerization, solution blending, or physical crosslinking [18]. These CHGs combine the softness and high water content of traditional hydrogels with nanomaterial‐driven enhancements, making them ideal for drug delivery, tissue engineering, PTT, and biosensing [19].

To overcome these limitations, recent studies have developed composite hydrogel systems by incorporating 2D materials (2DMats), particularly graphene, into hydrogel networks [20, 21]. These hybrid systems enhance electrical conductivity, mechanical strength, and biochemical functionality while preserving the tunable, water‐rich nature of hydrogels [22, 23]. Since its discovery in 2004, graphene has gained significant interest across biomedicine, energy storage, and electronic sensing due to its exceptional mechanical strength (up to 130 GPa), electrical conductivity (∼6000 S/cm), and thermal properties [24, 25]. These attributes have driven innovations in supercapacitors, batteries, photodetectors, and biointerfaces [26, 27]. Graphene‐based CHGs show particular promise in biomedical applications [28, 29]. Other 2DMats, such as transition metal dichalcogenides (TMDs), mono‐elemental xenes (e.g., arsenene, antimonene, phosphorene), MXenes, carbon nitrides, metal borides (MBenes), and boron nitrides, are also under investigation [30, 31, 32]. However, graphene remains the most versatile for biomedical hydrogels due to its superior electrical conductivity, mechanical strength, biocompatibility, and photothermal (PT) properties [33, 34]. Future research may explore hybrid systems combining graphene with MXenes or TMDs to further enhance hydrogel performance [34].

Graphene, a 2D carbon allotrope, is ideally suited for CHG development due to its high mechanical strength, tunable surface chemistry, and excellent thermal and electrical conductivities [35, 36]. Within hydrogels, graphene serves as a crosslinker or reinforcing filler, improving electrochemical response, structural durability, and stimulus sensitivity, critical for advanced biomedical applications [29, 37, 38]. The properties of CHGs depend on factors such as exfoliation techniques, lateral sheet dimensions, and graphene stability [39]. Exfoliation methods, which isolate single‐ or few‐layer graphene sheets, use external forces to disrupt non‐covalent interactions [40]. Chemical exfoliation alters graphene's surface chemistry, whereas physical exfoliation largely preserves its structural integrity and intrinsic properties [40, 41]. Optimizing these factors is essential for applications like light‐triggered drug delivery, which leverages near‐infrared (NIR) light for precise therapeutic release [42].

While numerous reviews have explored CHGs for biomedical applications, most lack a cohesive analysis of structure–function–application relationships. For example, Phan et al. and Santos et al. focused on conventional graphene–hydrogel composites and fabrication methods but provided limited insights into how nanoscale design influences biomedical outcomes [43, 44]. Similarly, Ni et al. examined graphene derivatives in biomedical systems but overlooked key translational challenges such as scalability, reproducibility, and long‐term biocompatibility, as well as associated design barriers [45]. Sharma et al. emphasized sustainable synthesis yet neglected digital optimization tools and stimuli‐responsive systems [46]. Huang et al. offered a broad classification of 2DMat‐based hydrogels but lacked detailed analysis of graphene's tunability for specific applications [47]. This review addresses these gaps by providing a comprehensive, forward‐looking analysis of the synthesis, functionalization, and biomedical applications of graphene‐based hydrogels. It further integrates ML as an emerging tool for rational design, predictive modeling, and performance optimization of CHGs. By bridging materials science and computational intelligence, this work establishes a roadmap for developing next‐generation, application‐specific smart hydrogels for precision drug delivery, tissue engineering, and PTT, setting a new standard for innovation in graphene‐integrated biomedical hydrogels.

## 2 Graphene Structure and Properties

**FIGURE 1:**
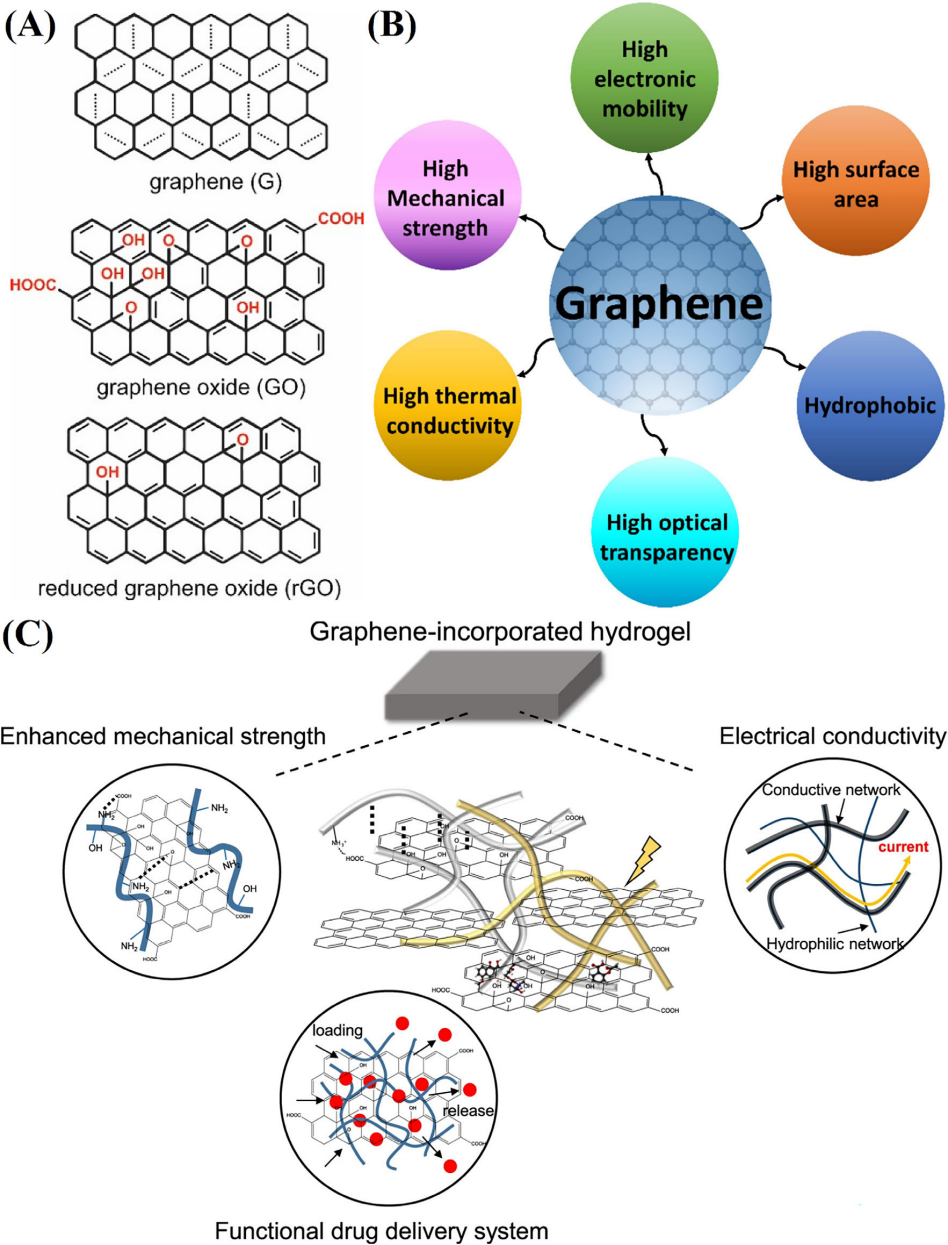
(A) Structures of graphene, GO, and rGO. Reproduced with permission [52]. Copyright 2018, MDPI. (B) General properties of graphene. Reproduced with permission [53]. Copyright 2024, Elsevier B.V. (C) Schematic representation of a graphene‐integrated hydrogel and its unique properties that facilitate the development of advanced functionalities. Reproduced with permission [54]. Copyright 2020, Springer Nature Limited.

Graphene, a single‐layer 2D carbon allotrope, consists of a densely arranged hexagonal lattice of sp^2^‐hybridized carbon atoms in a planar sheet [48]. Each carbon atom forms strong σ bonds with three neighboring atoms, with a bond length of 0.142 nm [49]. Its unique structure enables exceptional properties, making it ideal for integration into CHG matrices for biomedical applications. Graphene exists in multiple forms, including pristine graphene, graphene oxide (GO), reduced graphene oxide (rGO) (Figure 1A), graphene quantum dots (GQDs), few‐layer graphene, and functionalized derivatives, each tailored for specific applications [50]. High‐yield production of graphene derivatives typically involves oxidizing graphite to form GO, followed by chemical or thermal reduction to rGO, or using liquid‐phase exfoliation of graphite to obtain few‐layer graphene [51]. These structural features facilitate tailored polymer–graphene interactions, enhancing CHG performance in drug delivery, tissue engineering, and PTT, as illustrated in Figure 1C.

### 2.1 Mechanical Properties

Graphene exhibits an elastic modulus of approximately 1 TPa and a tensile strength around 130 ± 10 Gpa, making it one of the strongest materials [55]. In CHGs, high water content (often > 90%) can reduce mechanical strength, potentially weakening polymer–graphene interactions and compromising structural integrity [56]. However, incorporating oxygen‐containing functional groups on GO, combined with graphene's large specific surface area (∼2600 m^2^/g), significantly enhances hydrogel mechanical properties [57]. GO‐reinforced PVA hydrogels show a Young's modulus increase from 10 kPa to 100–200 kPa. Hydrophilic polymer chains form hydrogen bonds with GO's oxygen groups, strengthening the network [58]. During deformation, these bonds dynamically break and reform, enabling self‐healing and adaptability, critical for 4D‐printed dynamic scaffolds. Graphene's high fracture toughness further improves CHG resilience, supporting applications like cartilage repair. These enhancements make graphene‐based CHGs ideal for mechanically demanding biomedical platforms.

### 2.2 Biocompatibility

The biocompatibility of graphene and its derivatives strongly depends on factors such as concentration, lateral size, surface functionalization, dispersion state, and exposure duration [59]. Although many in vitro studies report low cytotoxicity for properly functionalized graphene materials across various cell lines, higher concentrations or prolonged exposure may reduce cell viability [60, 61]. Moreover, evidence suggests that polymeric and biomaterials metrices can mitigate the toxic effects of fillers. A major limitation of graphene is its tendency to agglomerate and revert to graphite, driven by strong van der Waals forces [62]. In fact, graphene shows poor dispersibility in both organic and aqueous solutions, especially those containing salts, proteins, or other ions [63]. Functionalizing graphene allows for stable suspension production, resolving this issue [51].

### 2.3 Photothermal and Electrical Properties

Graphene and its derivatives demonstrate outstanding electrical and PT properties, making them highly suitable for biomedical and energy applications [64]. Graphene's high thermal conductivity (typically 3000–5000 Wm^−1^K^−1^), ensures rapid heat transfer, enabling precise localized heating in PTT and bioelectronics [65]. Its semi‐metallic nature, high charge carrier mobility, zero‐bandgap structure, and near ballistic transport at nanoscale contribute to excellent electrical conductivity, facilitating applications in tissue regeneration, biosensors, and energy storage [66]. Additionally, highly rGO can achieve electrical conductivities of up to several thousand S/cm (e.g., ~1000–6600 S/cm in optimized thin films) [67, 68]. These combined properties of graphene and its derivatives highlight their versatility in enhancing the performance of CHGs for biomedical and technological advancements. Different general properties of graphene are illustrated in Figure 1B.

The integration of graphene into polymeric hydrogel networks can lead to changes in its intrinsic properties [68]. Electrical conductivity may be reduced, particularly with covalent functionalization or GO‐based systems, due to disruption of the sp^2^ lattice [69]. However, PT characteristics are typically retained or enhanced through uniform dispersion of rGO or GO [70]. Mechanically, the incorporation of graphene significantly strengthens the hydrogel due to its high tensile strength and stiffness [71]. Biocompatibility can also improve when graphene is encapsulated in biocompatible polymers, mitigating cytotoxic effects and improving physiological compatibility. These synergistic interactions between graphene and the polymer matrix underpin the multifunctional behavior of composite hydrogels in biomedical applications [72].

Compared to other 2DMats (e.g., MXenes, TMDs, boron nitride), graphene remains the most versatile for hydrogel reinforcement due to its combined strength, conductivity, and chemical tunability. However, large‐scale biomedical translation is limited by batch‐to‐batch variability, agglomeration, and incomplete understanding of long‐term biosafety. Addressing these issues requires standardized synthesis, dispersion protocols, and comprehensive toxicological studies to fully exploit graphene's multifunctional advantages in hydrogel applications.

## 3 Functionalization of Graphene: Methods and Molecular Interactions in Polymeric Hydrogel Systems

Functionalization of graphene‐based CHGs, play a major role in improving their performance for biomedical applications [73, 74]. By modifying graphene through covalent and non‐covalent functionalization, its biocompatibility, solubility, drug‐loading capacity, and release efficiency can be significantly enhanced, reducing potential toxicity concerns [71]. Integrating functionalized graphene into polymer matrices makes these CHGs ideal for applications in drug delivery and tissue regeneration [69].

### 3.1 Non‐Covalent Functionalization

Non‐covalent functionalization refers to the attachment of certain molecules onto graphene's surface through physical interactions rather than chemical bonding [75]. This process is primarily driven by *π–π* interactions, allowing molecules like surfactants, small aromatics, porphyrins, and biomolecules such as DNA and peptides to be immobilized [76]. Non‐covalent functionalization relies on weak interactions, preserving the hexagonal lattice of graphene [77]. Aromatic molecules like pyridine, fluorinated benzene, quinoline, and anthracene are used as stabilizers to prevent restacking of graphene sheets in aqueous or organic dispersions [78]. These compounds adhere to graphene surfaces through *π–π* interactions, often aided by sonication. Pyrene, in particular, exhibits strong interactions with graphene. Various studies have explored the *π–π* interactions of aromatic molecules with rGO and utilized pyrene derivatives as stabilizers for graphene nanoplatelets [79].

### 3.2 Covalent Functionalization

Covalent functionalization is typically achieved by oxidizing graphite to form GO [80]. The oxidation changes the sp^2^ carbon lattice structure of graphene to sp^3^ carbon, significantly reducing electrical conductivity [81]. Common methods for covalent functionalization include binding organic functionalities, such as free radicals and dienophiles, and covalently modifying GO [82].

Free radical reactions are often used with graphene nanomaterial to adjust solubility or compatibility with other materials [83]. When diazonium salts or benzoyl peroxide are heated, highly reactive free radicals are produced, interacting with the sp^2^ carbon of graphene to form a covalent bond [84]. Diazonium compounds with electron‐donating or withdrawing groups are frequently used to alter transport in silicon after aryl assembly on device surfaces [85].

Dienophiles typically react with sp^2^ carbon atoms in graphene [86]. A successful reaction in this category is the 1,3 dipolar cycloaddition of azomethine ylides, commonly used in functionalizing carbon nanotubes and fullerenes [87]. This versatile reaction offers various applications and results in highly functionalized materials.

GO is often used as a precursor for producing graphene by oxidizing graphite, followed by exfoliation and reduction. However, even after reduction, residual oxygen groups and structural defects impact the properties of the resulting rGO, particularly its electrical and mechanical characteristics [88]. Methods such as the Hummers, Staudenmaier, or Brodie techniques can produce GO [89]. GO can then be converted to rGO by restoring the sp^2^ bond network via thermal, chemical, or electrochemical reduction methods [90]. Optimizing functionalization strategies is crucial for developing graphene‐based hydrogels with enhanced biocompatibility, mechanical resilience, and controlled stimuli responsiveness.

In addition to graphene, several other 2DMats, such as MXenes, TMDs, boron nitride, and carbon nitride, have been explored for hydrogel reinforcement. Each offers distinctive benefits, yet they also present unique challenges that limit their biomedical translation. To position graphene within this broader context, Table 1 provides a side‐by‐side comparison of graphene and these emerging 2DMats, highlighting their properties, advantages, limitations, and biomedical relevance. This comparison underscores why graphene remains the most versatile and widely studied candidate for multifunctional hydrogel systems.

**TABLE 1:** Comparison of graphene and other 2DMats in hydrogel applications, outlining key properties, advantages, limitations, and biomedical relevance.

2D Material	Key properties	Advantages in hydrogels	Limitations/Challenges	Applications	Reference
Graphene & Derivatives (GO, rGO, GQDs)	High electrical & thermal conductivity, large surface area, tunable functional groups	Mechanical reinforcement, PT conversion, pH/NIR responsiveness	Tendency to agglomerate, potential cytotoxicity at high dose, batch variability	Drug delivery (DOX, 5‐FU), PTT, bone/cartilage scaffolds	[91, 92, 93]
MXenes (Ti_3_C_2_Tx, Nb_2_C, etc.)	Hydrophilic surface, high conductivity, rich surface chemistry	Excellent dispersibility in aqueous hydrogels, excellent electrochemical sensing	Prone to oxidation, limited long‐term stability in biological fluids	Electrochemical biosensors, wound healing monitors, tissue engineering	[94, 95, 96]
TMDs, e.g., MoS_2_, WS_2_	Semiconducting, strong light absorption, flexible nanosheets	PT and photocatalytic properties, tunable bandgap	Lower conductivity vs. graphene, potential long‐term toxicity	PTT, antibacterial hydrogels, nerve regeneration	[97, 98, 99]
Hexagonal Boron Nitride (h‐BN, BN nanosheets)	Chemically stable, high thermal conductivity, insulating	Biocompatibility, lubrication in cartilage‐like hydrogels	Electrically insulating, limited drug‐loading capacity	Joint lubrication, protective scaffolds, dental coatings	[100, 101, 102]
Carbon Nitride (g‐C_3_N_4_)	Visible‐light photocatalytic activity, semiconducting with low‐to‐moderate conductivity	Supports photocatalytic ROS generation, antibacterial activity	Weaker mechanical reinforcement compared to graphene	Antibacterial hydrogels, nerve conduits, photocatalytic wound dressings	[103, 104, 105, 106]

### 3.3 Computational Modeling of Graphene‐Polymer Interactions

Computational approaches, particularly density functional theory (DFT), have played a key role in revealing how graphene derivatives such as GO interact with polymer chains within CHGs. These studies provide clear insight into the molecular mechanisms that underlie improvements in mechanical strength, electrical performance, and biomedical functionality. The resulting models quantify critical parameters such as binding energies, charge transfer, and electronic descriptors, and these molecular‐level metrics correlate with enhanced durability and overall performance and provide indirect insight into biocompatibility in applications such as tissue engineering and drug delivery.

Adekoya et al. performed DFT calculations using the B3LYP functional with DNP basis sets on GO–PEG NCs [107]. Their results showed that the system adopts a stable configuration governed primarily by hydrogen bonding, with a gas‐phase binding energy of −25.67 kcal/mol between the oxygen of the PEG hydroxyl group and the hydrogen of the GO carboxylic group. Incorporating solvation effects through the COSMO model, using a dielectric constant of 78.54 for water, modified the interaction profile in in a manner consistent with controlled drug release. This behavior was evident in GO–PEG complexes with cephalexin, which exhibited binding energies of −36.17 kcal/mol in the gas phase and −26.38 kcal/mol in solvent, indicating favorable interactions that facilitate faster drug release in moist environments relevant to wound healing applications. Charge transfer analysis based on Mulliken population analysis suggested electron donation from the drug to the GO/PEG matrix of up to −0.117 e, confirming complex stability while preserving the drug's therapeutic functionality. These combined features position GO–PEG hydrogels as promising platforms for antimicrobial drug delivery in biomedical settings [107].

Similarly, Quanguo et al. performed DFT calculations on GO‐reinforced thermoplastic polyurethane (TPU) NCs and reported strong interfacial interactions, with binding energies of −35.303 kcal/mol at GO edges and −39.098 kcal/mol on GO surfaces. Atoms‐in‐molecules (AIM) analysis confirmed the presence of multiple hydrogen bonds. Reactive molecular dynamics (ReaxFF) simulations further showed significant charge transfer (3.483 e from TPU matrix to GO), which contributed to a 34% increase in Young’s modulus (from 281 GPa to 378 GPa in the idealized molecular model) at 18 wt% GO. These enhancements demonstrate the suitability of GO–TPU systems for flexible hydrogel composites intended for orthopedic scaffolds or injectable biomaterials that require high mechanical stability [108].

Further, Elhaes et al. conducted DFT modeling at the B3LYP/6‐31G(d,p) level of theory to investigate the interactions between lithium‐functionalized graphene and sodium alginate (SA) biopolymers [109]. Their results showed weak non‐covalent interactions that reduced the HOMO–LUMO energy gap to 0.280 eV and increased the dipole moment to 15.509 Debye in the optimized graphene/3SA/Li composites. These electronic changes increased the electrophilicity index (up to 71.963) and overall reactivity, thereby supporting improved electronic compatibility within hydrogel matrices for electrically conductive biomedical applications, including tissue regeneration and sensor‐integrated scaffold systems.

Although this review focuses on graphene, similar DFT observations have been reported for other carbon allotropes. Studies involving carbon nanotubes (CNTs) and fullerenes show comparable hydrogen bonding and charge transfer mechanisms that improve nanocomposite (NC) toughness, although graphene's planar geometry generally provides superior dispersion and interfacial contact in hydrogel systems. Taken together, these computational insights guide the rational design of multifunctional graphene–polymer hydrogels by optimizing interfacial interactions to achieve targeted biomedical functions such as sustained drug release and enhanced mechanical reinforcement [19, 110].

## 4 Synthesis Methods of Graphene‐Based CHGs

The performance of graphene‐based CHGs depends on the synthesis method [111]. Graphene dispersion, interfacial strength, component affinity, and spatial organization within the polymer matrix directly impact stiffness, strength, toughness, and elongation [71]. The synthesis of graphene‐based CHGs is influenced by various factors, such as the polarity, molecular weight, hydrophobicity, and surface chemistry of graphene, along with the reactive functional groups available in the hydrogel matrix, graphene itself, and the surrounding solvent [112]. Widely used techniques for fabricating these materials include blending in solution, thermal melt processing, and polymerizing the components directly within the system [113, 114, 115].

It is important to note that not all polymers are inherently compatible with graphene or its derivatives. The successful fabrication of graphene‐based CHGs depends on several factors, including the chemical structure, polarity, functional group availability, and molecular weight of the polymer [116]. Graphene's tendency to aggregate, driven by strong pi–pi interactions and van der Waals forces, often results in poor dispersion within polymer matrices that lack sufficient interfacial interactions. Hydrophobic polymers, for example, may require surfactants or functionalization strategies to improve compatibility. Similarly, polymers without reactive functional groups may fail to establish stable networks with graphene sheets, leading to phase separation or compromised mechanical and electrical performance. Therefore, the choice of polymer must be carefully tailored based on its chemical affinity with graphene and the intended biomedical application. In some cases, chemical modifications of either the polymer or graphene may be necessary to achieve uniform dispersion and enhanced composite properties [117].

### 4.1 Solution Mixing

This widely used method separates graphene sheets efficiently [118]. It involves three steps: dispersing the filler in a suitable solvent (e.g., via ultrasonication), incorporating the polymer, and removing the solvent (by distillation or evaporation) [71]. Graphene sheets are coated with polymer, and upon solvent evaporation, they interconnect with the polymer to form NCs [119]. Compatibility between polymer, filler, and solvent is essential for optimal dispersion. Common solvents include water, acetone, chloroform, tetrahydrofuran, dimethylformamide, and toluene [69]. This method is advantageous due to its simple, fast fabrication process, high control over component behavior, and effective filler dispersion [47, 112]. The main disadvantages include solvent toxicity, challenges in removing residual solvents, and NC aggregation [120].

### 4.2 Melt Blending

In this method, polymer melt and powdered graphene are mixed using high shear in a screw extruder or blender, without solvents [121]. Liquefying the polymer phase with high temperatures facilitates the dispersion and intercalation of GO and reduced graphene. This technique can be applied to both polar and nonpolar polymers; however, dispersion is generally more effective in polar systems and the method is cost‐effective and scalable for industrial use. However, the higher viscosity of hydrogel composites with increased graphene loading reduces the effectiveness of melt mixing in dispersing graphene sheets [71, 122].

### 4.3 In Situ Polymerization

This method requires monomers, an initiator, and a high‐temperature reactor. Polymerization starts with heat or radiation once the initiator has been diffused [123]. This approach facilitates more uniform graphene nanofiller distribution within the polymer matrix, enabling covalent bonding through various condensation reactions. However, this process is limited by the high costs of the monomer and a controlled reactor environment, often involving elevated temperatures or radiation sources [123].

## 5 Multifunctional Applications of Graphene‐Based CHGs

**FIGURE 2:**
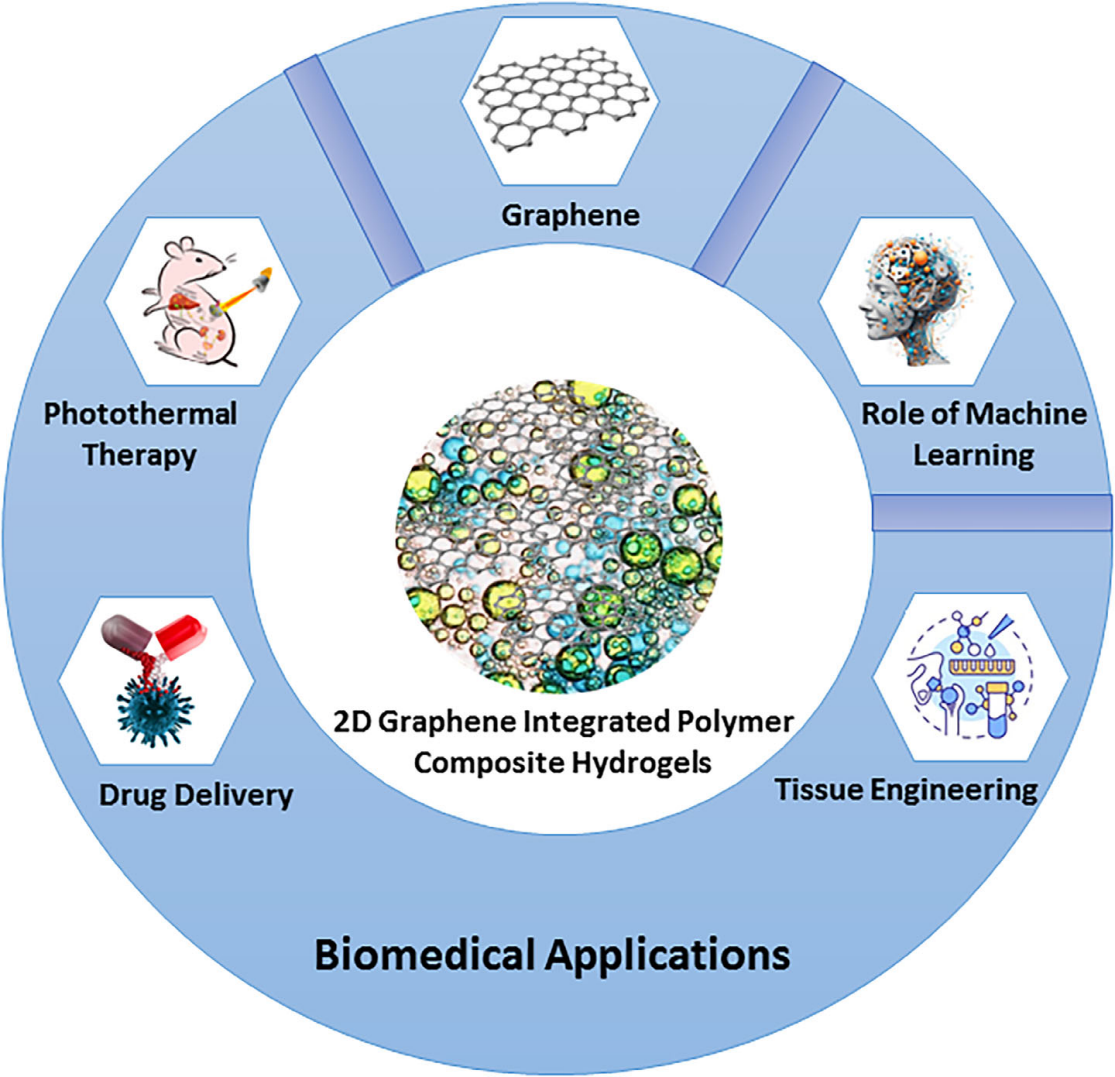
Graphene integrated polymer‐based CHGs for various biomedical applications and the role of ML.

The unique 2D planar structure and diverse chemical compositions of graphene and its derivtives have lead to unique properties of CHGs for biomedical applications [120]. To date, these graphene‐based CHGs have been explored for various biomedical applications [124]. We have summarized and critically analyzed the recent advances in 2D graphene‐based CHGs for applications in drug delivery, tissue engineering, and PTT, as illustrated in Figure 2 and summarized in Tables 2, 3, and 4.

**TABLE 2:** Summary of graphene‐based CHGs for drug delivery applications.

Applications	Material composition	Drug	Drug release mechanism	Manufacturing method	Reference
DOX delivery for treatment of blood cancer cells (K562)	CMCel/GQDs NC	DOX	pH sensitive swelling & diffusion	Solvent Casting	[125]
Targeted chemotherapy for HT‐29 colon cancer cells	CS/rGO NC	5‐FU & CU	Controlled drug release study via kinetic models	Chemical crosslinking	[126]
Diabetes treatment and HepG2 cell line liver‐targeted therapies	GO + MH hydrogel	MH	Diffusion‐controlled with swelling‐induced release and electrostatic interactions between MH and GO at pH 7.4	Physical crosslinking	[127]
DOX delivery for SW480 human colon cancer cells therapy	CMCel/GO	DOX	pH‐dependent release, *π–π* stacking interaction, and H‐bonding	Physical crosslinking	[128]
CU delivery for prostate cancer cell line PC‐3 treatment	CS/GO NC	CU	pH‐sensitive release (faster at acidic pH)	Spray drying	[129]
Load‐bearing cartilage repair/replacement, severe arthritis treatment	Glycerol‐modified PVA hydrogel; 3D PCL‐Graphene	FL	On‐demand drug release and PT conversion.	3D Printing and Freeze‐Thaw Crosslinking	[130]
Cancer treatment	Go/CMCh	QCT, GA	Sustained release under physiological pH	Physical crosslinking	[131]
Lung cancer therapy	GO/CS/HA	SNX‐2112	pH‐responsive release	Physical crosslinking followed by chemical cross linking	[132]
Oral drug delivery	CS/GO	MTD	Prolonged release in Simulated gastric fluids	Chemical cross linking	[133]
Gastric adenocarcinoma cell line therapy	CMCH/Gel, PEG/GO	Berberine chloride	pH‐responsive release	Self‐crosslinked via EDC and NHS chemistry	[134]

**TABLE 3:** Summary of graphene‐based CHGs for tissue engineering.

Applications	Polymer/Monomer	Active or Drug ingredient	Manufacturing method	Reference
Bone tissue engineering	Maleilated chitosan/ L‐cysteine,	BMSCs	click chemistry	[135]
Bone regeneration	Alg, Sericin,	Sericin, GO, BMSCs	Enzymatic Crosslinking	[136]
Calvarial defect repair	Gel	Methyl Vanillate	Photo‐crosslinking	[137]
Restoration of articular cartilage defects	GG/PEGDA	GO as dopant for lubrication enhancement	Photo‐initiated and ionic crosslinking	[138]
TMJ disc replacement for long‐term support	PU/PVA	nGO‐APU/PVA	Freeze thaw, Annealing	[139]
Orthopedic implants/artificial joints	Cyclodextrin‐based pseudopolyrotaxane hydrogel	Vancomycin	Physical & chemical crosslinking	[140]
Load‐bearing cartilage repair, Arthritis treatment	PVA /PCL	Glycerol‐modified PVA hydrogel; 3D PCL‐Graphene	3D Printing & Freeze‐Thaw Crosslinking	[130]
Cartilage repair	GEH	GO	Freeze casting	[141]
Neural tissue engineering	Col/CS	CSGO	Physical crosslinking	[142]
Neural tissue engineering	OPF/pega	GOa/ CNTpega	Chemical crosslinking	[143]
Peripheral nerve regeneration	g‐C_3_N_4_	g‐C_3_N_4_/GO	Chemical crosslinking	[144]
Tendon‐bone interface (TBI) healing,	PRP gel	Platelet‐derived growth factor (PDGF‐AB), Transforming growth factor‐β1 (TGF‐β1)released by PRP, GO	Physical crosslinking	[145]
Healing of injured Achilles tendon	Gel/ Polyglycerol	PG/ PMoS_2_	Chemical crosslinking	[146]
Tissue regeneration for rotator cuff repair in a rat model	NaAlg	GO/NaAlg	Chemical crosslinking	[147]

**TABLE 4:** Summary of graphene‐based CHGs for PTT.

Applications	Polymer/Monomer	Active or Drug ingredient	Manufacturing method	Reference
Multimodal Cancer Therapy	SE hydrogel	rGO, AuNCs, 5‐FU	Green synthesis, Self assembly	[148]
Tumor Imaging & PTT	SF hydrogel	UCNPs, NGO	Ligand exchange, self assembly	[149]
Chiral Drug Delivery, Chemotherapy & PTT	DPFEG	Oxaliplatin	Co‐assembly	[150]
Postoperative Recurrence Prevention in Breast Cancer, PTT	CSMA, BPEI	DOX	Schiff‐Base Linkage	[151]
Chemo‐PTT	CMC, CHO‐PEG	DOX	Chemical & thermal reduction	[152]
Colorectal cancer treatment, NIR‐triggered therapy	HA/ Polyaspartamide	Irinotecan	Physical assembly	[153]
Breast cancer treatment, Chemo‐PTT, Antibacterial applications	CS, Agarose	DOX, Ibuprofen	Physical assembly	[154]
Intratumoral drug delivery, chemo‐PTT for breast cancer	CS, Poloxamer 407, Poloxamer 188	DTX	Physical self‐assembly	[155]
Post‐surgical melanoma treatment, chemo‐PTT	ChiMA, SISMA	rGO‐DOX	Photo polymerization	[156]
Tumor inhibition & PTT	PEG, CMC	GO, HAP	Schiff base reaction	[157]

### 5.1 Drug Delivery

The conventional method of drug administration often requires large doses or frequent use to fulfil the required therapeutic effect. This approach can reduce overall efficacy, lower patient adherence, and increase the risk of side effects [158]. On the other hand, hydrogel‐based drug delivery platforms have demonstrated encouraging outcomes in clinical applications [72, 159]. Hydrogels serve as flexible platforms for controlled drug release. They can be easily modified, naturally biodegrade, and protect drugs from degradation [160]. Their mechanical strength allows them to withstand handling during production, implantation, and drug delivery in appropriate formulations. A robust structure ensures the hydrogel remains intact, improving drug delivery efficiency. To enhance the mechanical properties of polymer‐based hydrogels, various strategies have been employed. These include altering polymer composition, adjusting crosslinking density, and incorporating fillers or 2DMats like graphene. Such modifications help control drug release rates, improve tissue compatibility, and improve therapeutic effectiveness [161, 162, 163].

Since their introduction as bio‐secure materials, graphene NCs have been widely explored as molecular carriers for drug delivery [113]. Their broad specific surface area allows them to deliver multiple drugs to targeted locations from the point of administration. The functionalization of graphene improves its properties for biomedical applications. Covalent and non‐covalent modifications enhance biocompatibility, solubility, and drug‐loading capacity [74]. PEGylation extends circulation time and reduces toxicity [164]. CS coating improves mucoadhesion, making graphene suitable for oral and transdermal drug delivery [165]. Polymeric conjugation enables targeted drug delivery through ligand‐receptor interactions [166]. Graphene's aromatic structure allows it to interact with hydrophobic drugs via *π–π* stacking. This enables stable encapsulation of therapeutic agents such as docetaxel (DTX) and doxorubicin (DOX) [167]. As a result, graphene‐based systems offer high drug‐loading capacity, prevent premature release, and enhance therapeutic efficacy [168]. Many graphene‐based CHGs exhibit pH‐responsive behavior. This ensures site‐specific drug release under physiological conditions [169]. For example, in acidic tumor environments, DOX‐loaded GO hydrogels release the drug due to protonation‐induced structural changes [170]. Similarly, NIR light can induce PT heating, accelerating drug diffusion and enabling on‐demand release [171]. Graphene‐based CHG drug delivery systems can outperform conventional carriers such as liposomes and polymeric micelles in key aspects, including higher drug loading efficiency, improved stability, and enhanced controlled release capabilities [71, 172, 173].

**FIGURE 3:**
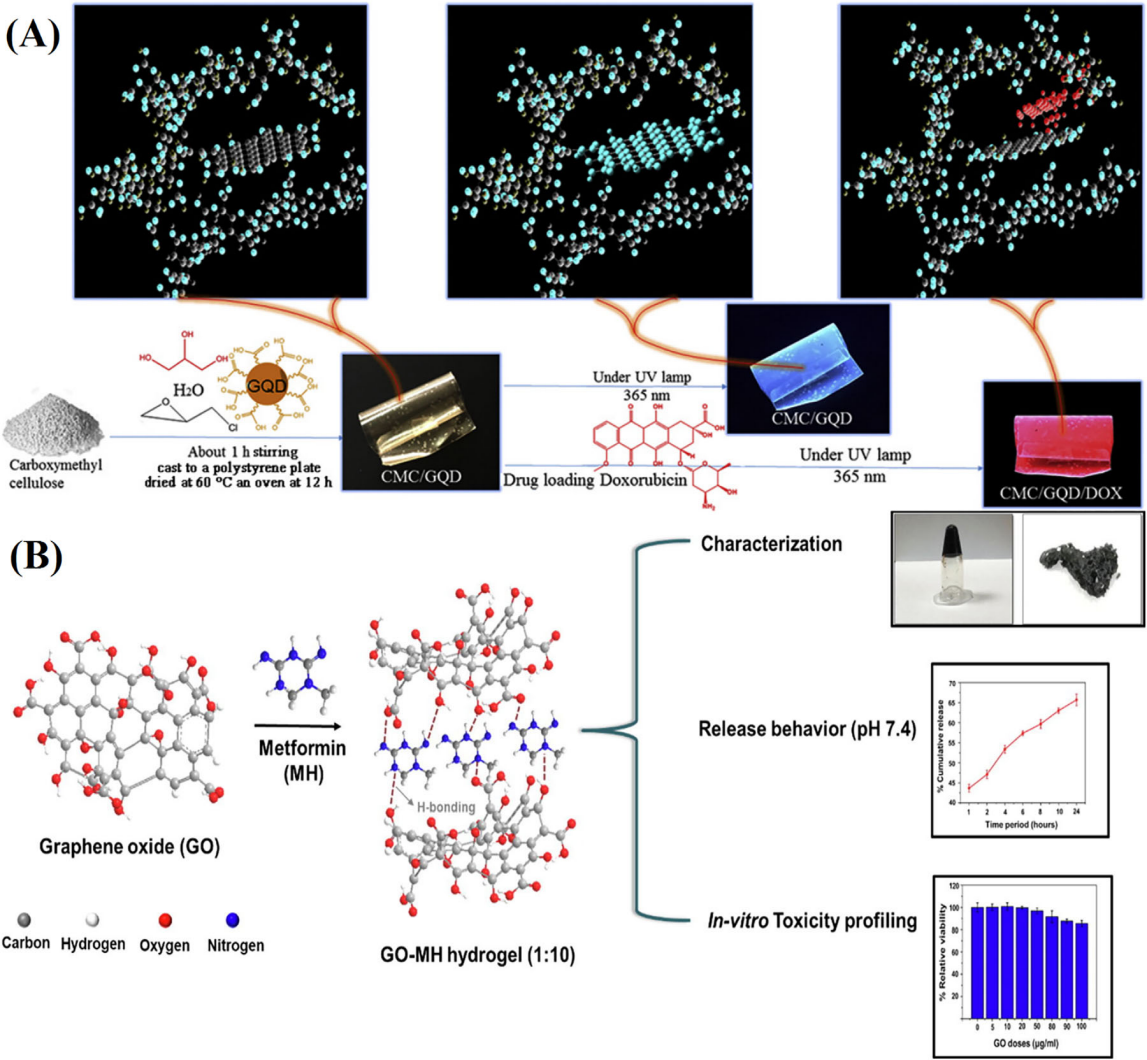
(A) General illustration of the synthesis of CMC/GQD and drug loading CMC/GQD/DOX. Reproduced with permission [125]. Copyright 2018, Elsevier B.V. (B) Illustration of GO‐MH hydrogel fabrication, characterization, release behavior, and in vitro toxicity profile. Reproduced with permission [127]. Copyright 2021, Elsevier B.V.

DOX is a widely used anticancer drug in chemotherapy. It works by incorporating into DNA, preventing cell division and DNA replication, which ultimately kills cancer cells [174]. However, current delivery methods, such as liposomes, nanoparticles, and NCs, face limitations when administered through blood vessels, including rapid clearance, poor tumor penetration, and potential off‐target toxicity [175]. A more effective approach is needed, one that allows direct interaction between the drug carrier and cancer cells. To address this issue, Javanbakht et al. synthesized hydrogel NC films with anticancer properties. They incorporated GQDs into carboxymethyl cellulose (CMCel) hydrogel and used DOX as a model drug, as shown in Figure 3A. Their study revealed that free DOX exhibited higher cytotoxicity than DOX/CMCel/GQD NCs. This difference was due to strong *π–π* stacking, hydrogen bonding, and electrostatic interactions between DOX and GQDs. These interactions led to a slow and steady release of DOX from the hydrogel. Further experiments evaluated drug release at different pH levels and tested the hydrogels against blood cancer cells (K562) using MTT assays. The results showed that these NC films had improved swelling, degradation, and water vapor permeability. They also exhibited pH‐sensitive drug release and controlled cytotoxic effects to K562 cells. Compared to conventional CMCel hydrogels and other carriers like carbon nanotubes and polymeric micelles, GQD‐based carriers demonstrated superior DOX loading and delivery efficiency. These findings suggest that CMCel/GQD NC hydrogel films have strong potential as anticancer agents and effective drug delivery systems [125].

Building on this, graphene, particularly in the form of GO and rGO, has emerged as a promising platform for drug and gene delivery. Its oxygen‐containing functional groups and high surface area provide excellent drug‐loading capacity, solubility, and biocompatibility [176]. Additionally, its inherent hydrophobicity makes it an ideal nanocarrier for cancer drugs. Considering these advantages, Dhanavel et al. developed a chitosan/reduced graphene oxide (CS/rGO) NC loaded with 5‐Fluorouracil (5‐FU) and curcumin (CU) using a simple chemical method. The nanocarrier exhibited high entrapment efficiency (> 90%) and efficiently suppressed the growth of HT‐29 colon cancer cells. However, its long‐term biocompatibility remains understudied. The combined cytotoxic effect of 5‐FU and CU‐loaded CS/rGO NCs resulted in an IC_50_ value of 23.8 µg/mL. Drug release was influenced by *π–π* interactions between rGO and CU, rGO and 5‐FU, and the interaction between 5‐FU and CU [126]. Future studies should focus on standardizing toxicity assays and exploring surface modifications to minimize potential adverse effects.

Extending this concept, Singh et al. developed a hydrogel composed of GO and metformin hydrochloride (MH) using a 1:10 metformin‐to‐GO weight ratio, as illustrated in Figure 3B. In vitro results showed a sustained metformin release of 67% at pH 7.4 over 24 h, demonstrating the hydrogel's potential for controlled drug delivery applications. These findings suggest that thebased hydrogel system exhibits acceptable in vitro safety,. In summary, this study offers novel insights into the in vitro therapeutic potential of the GO‐MH hydrogel [127].

**FIGURE 4:**
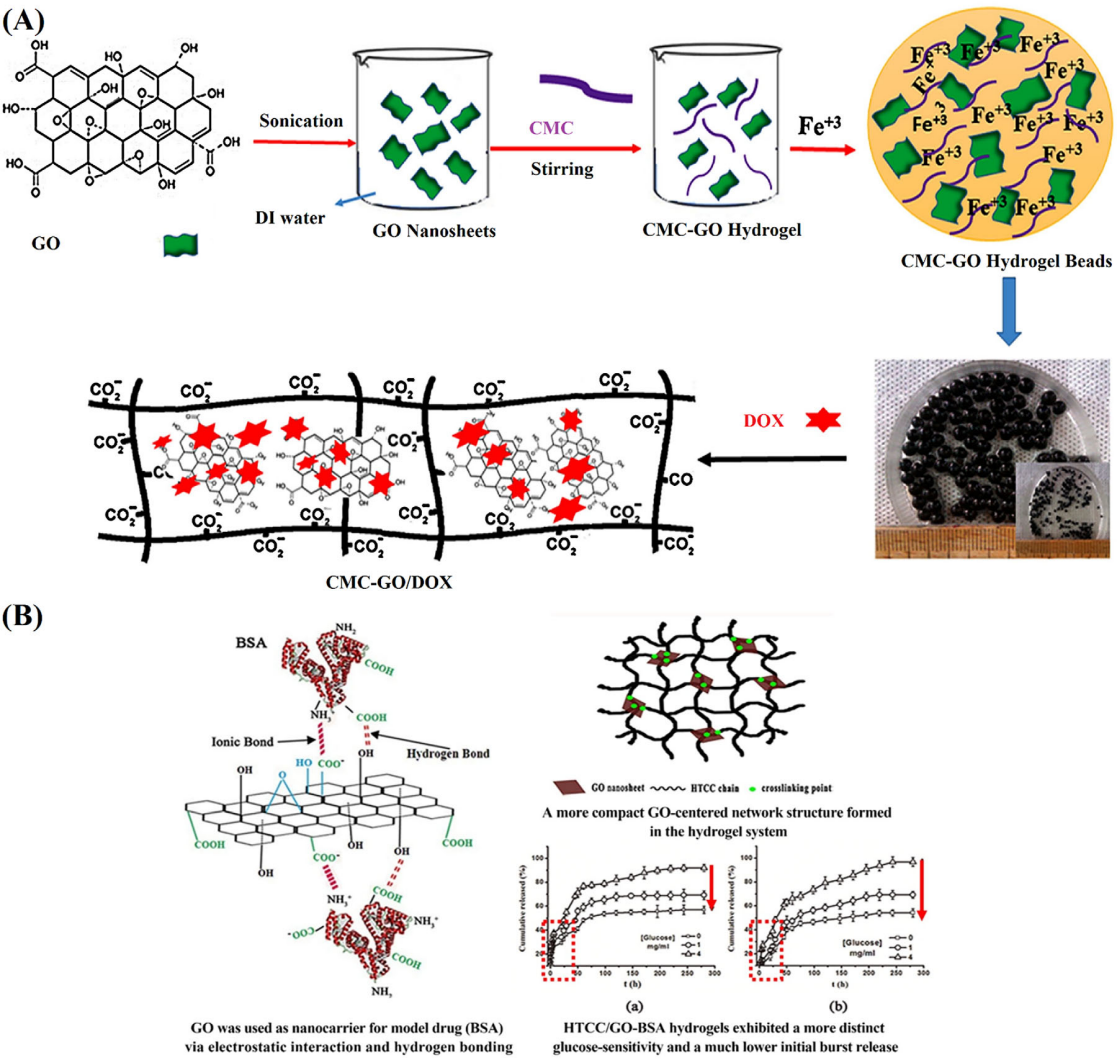
(A) Schematic diagram illustrating the synthesis process and chemical structure of the CMCel‐GO/DOX. Reproduced with permission [128]. Copyright 2017, Elsevier B.V. (B) Diagrammatic representation of general structure features of the HTCC/GO‐BSA hydrogel with drug release profiles. Reproduced with permission [177]. Copyright 2016, Elsevier B.V.

In a similar approach, Rasoulzadeh et al. developed hydrogel beads using CMCel for drug delivery. They utilized FeCl_3_.6H_2_O for physical crosslinking and controlled release of DOX, as shown in Figure 4A. The loading and release of DOX from the CMCel/GO‐CHGs were enhanced by *π–π* stacking interactions between DOX and GO. The drug release exhibited strong pH dependence at pH 6.8 and 7.4. In acidic conditions (pH 6.8), hydrogen bonds between GO and DOX were disrupted, leading to faster drug release. Drug loading efficiency increased with a higher concentration of nanoparticles. However, the release rate was higher in pure hydrogel than in NCHGs. As the GO content increased, drug release decreased due to strong interactions between the amine groups of DOX and the carboxyl groups of GO [128].

A ground‐breaking study reported that GO@DOX exhibited increased drug release at pH 5.3. This was due to a weakened interaction between DOX and the drug carrier [178]. Additionally, GO@DOX demonstrated enhanced cellular toxicity and significant tumor inhibition, inhibiting approximately 66%–91% of cancer cells [179]. Another study highlighted the anticancer efficacy of paclitaxel (PTX) and methotrexate (MTX) loaded onto GO via *π–π* stacking and amide bonding. This formulation effectively inhibited lung and breast tumor growth by 66%–90% [180].

Similarly, Zhao et al. developed NCHGs using cationic chitosan (HTCC) and GO as a nanocarrier for bovine serum albumin (BSA) delivery in hyperglycemia treatment, as shown in Figure 4B. Electrostatic interactions facilitated BSA entrapment within the GO layers, creating a composite with enhanced pH sensitivity and a reduced initial burst release. The swelling capacity of HTCC/GO‐BSA hydrogels declined with increasing GO‐BSA content. This effect was attributed to the increased crosslinking density and the tightly packed network structure observed in hydrogels containing higher concentrations of GO‐BSA [177].

**FIGURE 5:**
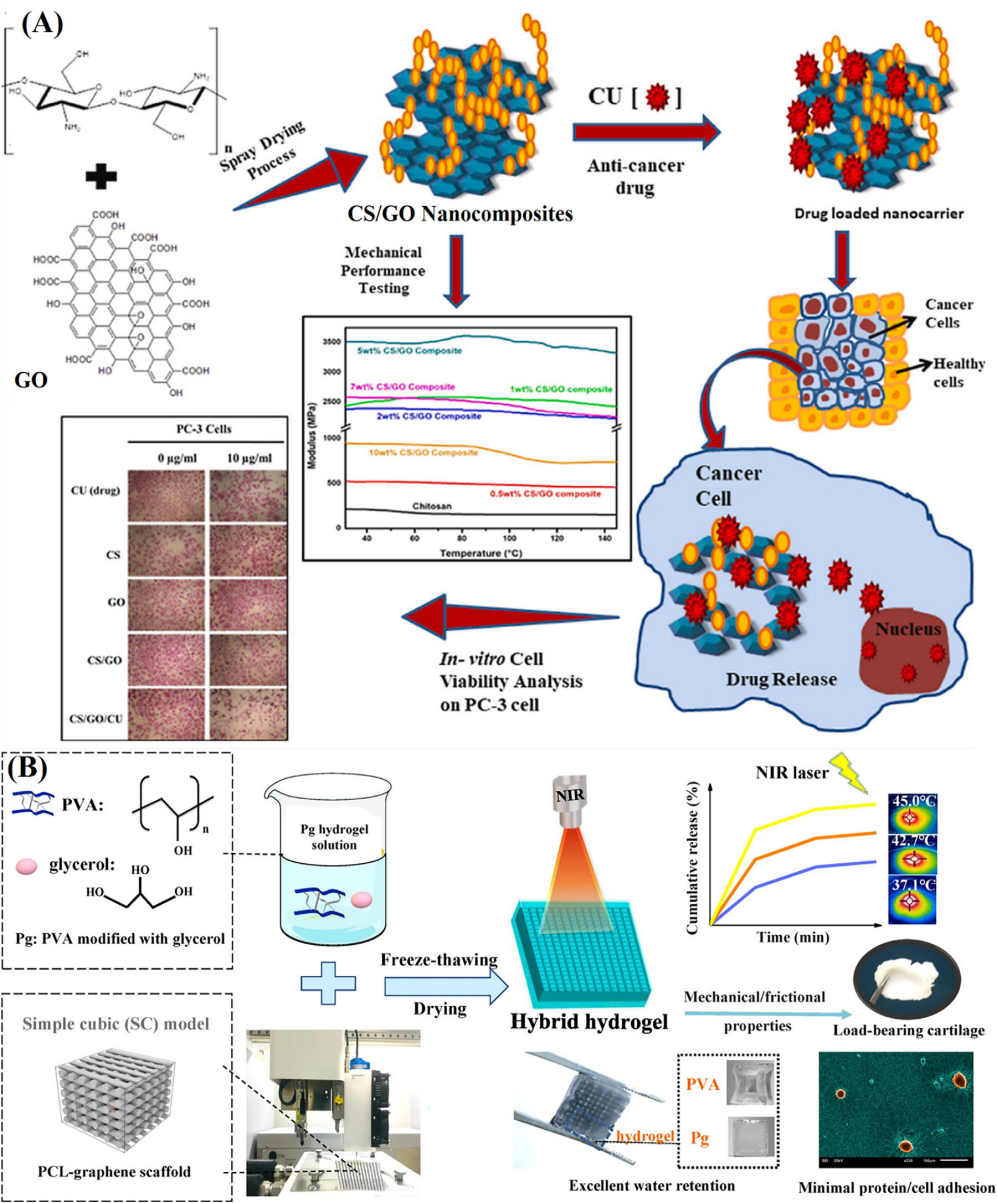
(A) Illustration of the synthesis of CS/GO NC and the CU drug loading and release behavior to kill cancer cells. Reproduced with permission [129]. Copyright 2022, Elsevier B.V. (B) Illustration of the fabrication and characteristics of the PG‐Pg hybrid hydrogel. Reproduced with permission [130]. Copyright 2020, Springer Nature Limited.

In a similar vein, Pandey et al. developed biocompatible CS@GO NCs using spray drying, a single‐step process that reduces particle size, modifies surface morphology, and converts liquid suspensions into dry powders with improved solubility, as illustrated in Figure 5A. The resulting CS/GO nanocarriers demonstrated a 74% CU drug entrapment efficiency, highlighting their potential for drug delivery applications. In vitro drug release studies showed a rapid release of approximately 35% of CU in acidic conditions (pH 4) and 16% at physiological pH (7.4) within 6 h, followed by a sustained release over 24 h. Additionally, cytotoxicity assays on human prostate cancer (PC‐3) cells indicated that CS/GO/CU NCs exhibited greater cytotoxic effects than free CU, emphasizing their enhanced therapeutic potential. These findings support the feasibility of spray‐dried CS/GO nanocarriers as a promising platform for controlled drug delivery [129].

Building on the concept of functional nanocarrier systems for drug release modulation, Jiang et al. developed a hybrid hydrogel system by modifying PVA with glycerol (Pg) and reinforcing it with a 3D‐printed poly(ɛ‐caprolactone) (PCL)‐graphene composite scaffold (PG). Since sodium fluorescein [85] was embedded only in the hydrogel component of the PG‐Pg hybrid system, it exhibited an initial burst release during the first 4 h, followed by gradual stabilization over 24 h. Increasing the glycerol content in the hydrogel led to a higher cumulative drug release. This was attributed to the faster molecular exchange enabled by the increased volume expansion of hydrogels with higher glycerol content. The drug release profile and elution mechanism of Pg hydrogels are illustrated in Figure 5B. Under NIR irradiation, PG‐Pg heated up, increasing molecular mobility, which accelerated water transport and drug diffusion, thereby enhancing drug release. By adjusting the laser power, researchers could control the cumulative drug release, demonstrating the potential for sustained and adjustable drug delivery through PT conversion [130].

**FIGURE 6:**
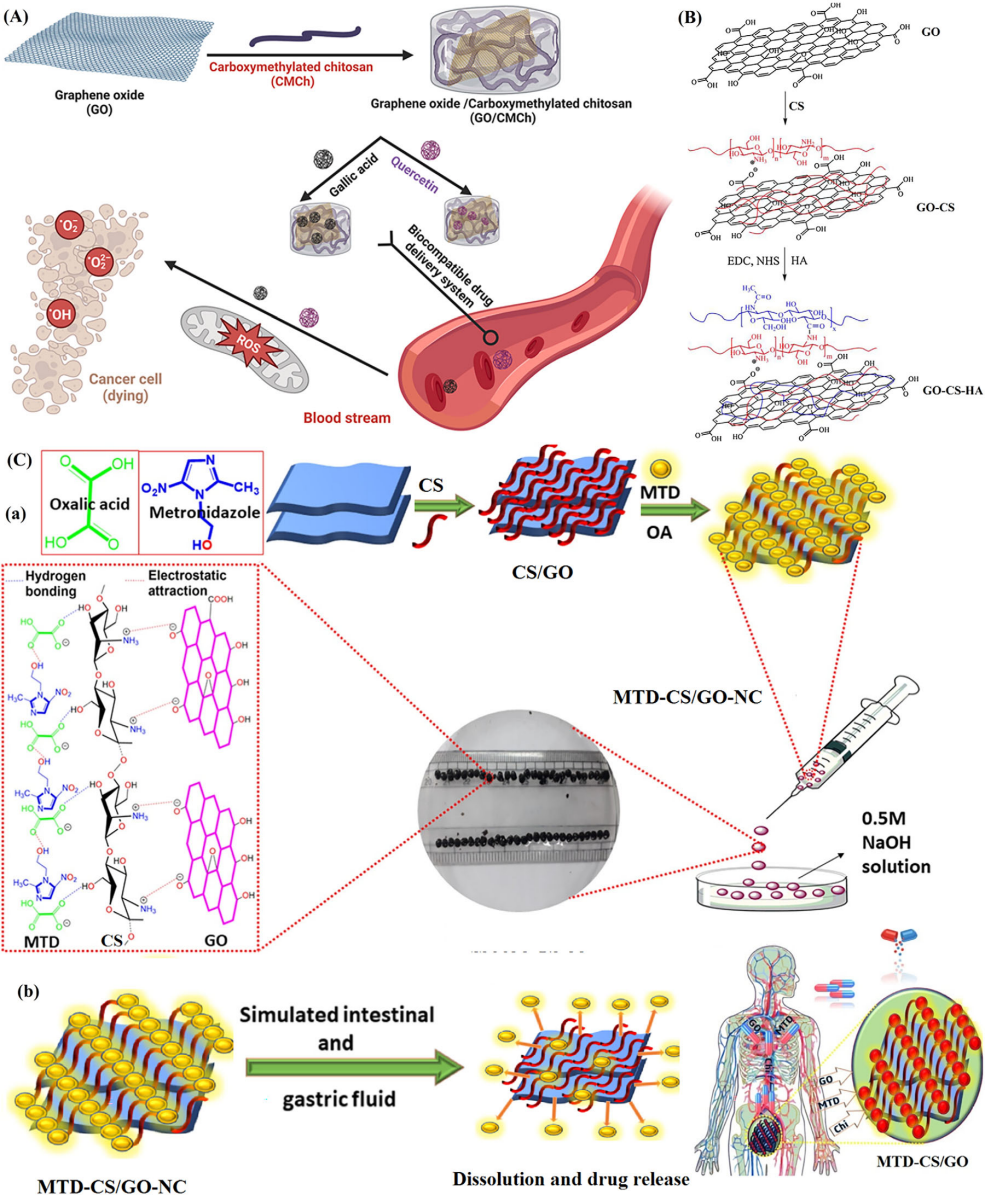
(A) General illustration of the GO/CMCh hydrogel for GA and QCT drug delivery for cancer treatment. Reproduced with permission [131]. Copyright 2024, Elsevier B.V. (B) Synthesis scheme of GO‐CS‐HA NC hydrogel. Reproduced with permission [132]. Copyright 2018, Elsevier B.V. (C) (a) Illustration of the preparation steps for MTD‐CS/GO bio‐NC. (b) Schematic illustration of drug release from the MTD‐CS/GO. Reproduced with permission [133]. Copyright 2021, American Chemical Society.

Expanding on the role of graphene‐based NCs in drug delivery, Salama et al. developed a GO/carboxymethyl chitosan (GO/CMCh) NC hydrogel, as shown in Figure 6A. Loading quercetin (QCT) and gallic acid (GA) onto the GO/CMCh hydrogel significantly improved their stability and therapeutic efficacy. The system achieved high entrapment efficiencies—98% for QCT and 99.3% for GA. Additionally, the loading capacity was remarkable: 200% for QCT and 250% for GA. This novel approach enhanced drug stability and targeted delivery, particularly for hepatocellular carcinoma therapy under normal pH conditions. The developed NCs also exhibited low hemolysis (below 1%), confirming their safety for clinical applications. These promising results suggest the need for further in vivo research on GA@GO/CMCh for cancer treatment and drug delivery [131].

In a similar effort to harness graphene‐based composites for targeted therapy, Liu et al. synthesized an innovative GO‐based drug carrier using CS and HA, as illustrated in Figure 6B. This system was designed to target tumor cells via CD44 receptors, thereby enhancing drug delivery. The researchers incorporated SNX‐2112, an Hsp90 inhibitor and anticancer drug, into the GO‐CS‐HA composite. Drug release studies showed that SNX‐2112 was released more rapidly under acidic conditions than at physiological pH, improving its tumor‐targeting potential. Toxicity assessments revealed that GO‐CS‐HA had minimal effects on red blood cell (RBC) lysis and blood coagulation, indicating low toxicity. Additionally, cytotoxicity studies on A549 (lung cancer) and NHBE (normal lung) cells demonstrated that GO‐CS‐HA/SNX‐2112 effectively inhibited A549 cell growth while causing minimal harm to NHBE cells. Further vital organ toxicity studies in Sprague Dawley (SD) rats, including histological and blood analyses, showed that GO‐CS‐HA/SNX‐2112 caused only short‐term inflammation with no severe long‐term injury. In conclusion, this GO‐based drug delivery system demonstrated strong therapeutic potential for cancer treatment due to its efficacy, safety profile, and minimal adverse effects [132].

Extending the application of graphene‐based NCs beyond cancer therapy, Kumar et al. synthesized CS/GO bio‐NC beads using a simple gelation approach. These beads were designed for the oral delivery of metronidazole (MTD), with drug release monitored over 84 h. As illustrated in Figure 5C, MTD was loaded both on the surface and within the cavity of the CS/GO beads, achieving an impressive drug loading capacity of 683 mg/g. To evaluate drug release performance, researchers analyzed the release profiles of pure MTD and CS/GO‐encapsulated MTD in simulated gastric (pH 1.2) and intestinal (pH 7.4) fluids using phosphate‐buffered saline (PBS). Results showed that the bio‐NC beads provided greater stability and a prolonged drug release profile compared to the pure drug. This enhanced bioavailability while minimizing potential side effects, making CS/GO beads a promising platform for oral drug delivery [133].

In response to the growing demand for biocompatible and safe hydrogel systems for biomedical applications, Shahzad et al. addressed the limitations of conventional organic crosslinkers—such as poor water solubility, slow biodegradation, and potential toxicity—by developing a self‐cross‐linked carboxymethyl chitosan (CMC) hydrogel (CMC HG) using EDC/NHS chemistry. This strategy eliminated the need for toxic organic agents and provided a fast, cost‐effective, and environmentally friendly alternative for hydrogel synthesis. To enhance physical and chemical properties, researchers incorporated gelatin Gel and 6‐arm PEG‐functionalized GO, forming CMC/GL HG and CMC/GL/PEG‐GO HG composites. In vitro studies revealed that CMC/GL/PEG‐GO HG released 30% of berberine chloride within 12 h and 39% cumulatively over 96 h, demonstrating controlled drug release. Furthermore, the self‐cross‐linked CMC hydrogel composite exhibited potent antioxidant and anticancer effects, along with biodegradability and low toxicity. These properties suggest that CMC/GL/PEG‐GO HG is a promising drug carrier for tumor therapy, potentially reducing chemotherapy side effects while improving therapeutic outcomes [134].

Overall, graphene‐based CHGs exhibit remarkable potential in drug delivery owing to their high surface area, tunable release profiles, and biocompatibility. The reviewed systems demonstrate effective controlled and site‐specific release of various therapeutics, including anticancer drugs such as DOX, 5‐FU, CU, QCT, GA, and SNX‐2112, as well as MTD and berberine. However, challenges such as toxicity concerns, stability issues, and the complexity of large‐scale fabrication hinder their clinical translation. While studies demonstrate controlled drug release and enhanced therapeutic efficiency, inconsistent in vivo results raise concerns about reproducibility. Moreover, interactions with biological systems require deeper investigation to minimize unintended cytotoxic effects. Future research should focus on optimizing functionalization strategies and ensuring long‐term safety. Addressing these limitations could pave the way for graphene‐integrated drug carriers in precision medicine. Thus, despite promising advancements, further refinements are essential for clinical success.

### 5.2 Tissue Engineering

Annually, millions of patients suffer from organ or tissue failure due to accidents or diseases. The global shortage of potential organ donors has sparked scientists to explore alternative solutions to meet the growing demand for organ transplantation [181]. Over the past two decades, considerable attention has been devoted to addressing this issue through the development and incorporation of biocompatible and stimuli‐responsive materials for tissue regeneration. Hydrogels have gained recognition as potentially effective candidates for mimicking various tissues in the body [182]. The process typically involves integrating cells into the hydrogel structure, followed by gradual degradation, which ultimately promotes the regeneration of healthy tissue [183]. Hydrogels have gained substantial recognition due to their excellent water retention, ability to maintain a porous structure, and adaptability to diverse sol‐gel conditions [184]. These unique structural properties make hydrogels suitable for use as tissue scaffolds, facilitating the exchange of cellular metabolites and the removal of waste through their pores [185]. The development of modified organs or tissues for patients requiring transplants is a pioneering approach that has led to the advancement of sophisticated hydrogel materials. This includes the creation of supramolecular, micro‐engineered, and graphene‐integrated NanoMats, opening new possibilities in tissue engineering and regenerative medicine.

#### 5.2.1 Hard Tissue Regeneration

Hard tissue regeneration, such as bone and cartilage repair, requires materials that provide mechanical support and promote cellular growth [186]. These tissues present unique challenges, including the need for strength and proper cellular alignment for effective regeneration [187]. To address these challenges, graphene nanosheets integrated with hydrogels are increasingly being utilized in tissue engineering and regenerative medicine [68]. Graphene‐hydrogel scaffolds have shown promising applications, ranging from periodontal to neural regeneration. Studies have shown that graphene‐enhanced scaffolds can promote osteoblast adhesion and mineral deposition, both of which are essential for bone regeneration, due to the combined benefits of graphene's mechanical strength and the biocompatibility and water‐retention capacity of hydrogels [188]. In tissue engineering, graphene‐integrated hydrogels offer distinct advantages over other materials [138]. For example, graphene provides greater mechanical strength and elasticity compared to collagen (Col) as a reinforcing nanofiller, which is commonly used in tissue engineering [189]. Graphene‐based scaffolds exhibit enhanced mechanical durability, supporting long‐term structural stability in vivo [190]. On the other hand, hydrogels establish a hydrated, ECM‐mimicking microenvironment [191]. By incorporating graphene into hydrogels, researchers can enhance the surface features and structure of these materials, enabling spatial and temporal control over cellular adhesion, proliferation, and alignment [192]. This approach enables cells to grow and align properly, facilitating the creation of small tissue like constructs that closely resemble their natural counterparts in both function and structure [193]. Such advanced scaffolds offer enhanced load‐bearing capacity and osteoinductive behavior, making them promising candidates for applications in cranial, dental, and orthopaedic bone repair [194].

Graphene composites can offer potential for the creation of multi‐functional composite inks, which can be tailored to exhibit properties such as biocompatibility, photoresponsiveness, electrical conductivity, and hydrophilicity [195]. The incorporation of graphene fillers improves the printing process by reducing the coefficient of friction, ensuring precise deposition with minimal defects [196]. By harnessing graphene's superior electrical properties, recent studies have focused on developing conductive bioinks for applications in nerve and muscle regeneration, as well as cardiovascular tissue engineering [197, 198, 199].

Advances in 3D bioprinting technologies have enabled the automated construction of complex tissue systems with precise control. Since tissue regeneration often involves structural changes over time, requires integrating dynamic adaptability into 3D‐printed constructs to mimic natural tissue development [200]. 4D bioprinting addresses this challenge by integrating graphene, which imparts electroconductive properties to the printed structures. As a result, graphene‐based nanomaterials are emerging candidates for bioinks in 4D bioprinting applications [201]. Furthermore, graphene‐based CHGs have shown significant potential in regenerating various tissues, including bone, cartilage, skin, and nerve tissue.

Bone, a vital bioceramic composite connective tissue, plays an essential role in supporting and protecting organs, producing blood cells, and storing minerals. Its intricate structural organization is crucial for its mechanical properties, material exchange, and cellular functions [202]. While bone has natural self‐healing abilities, these are often insufficient in complex clinical scenarios that require extensive regeneration [203, 204]. Traditional treatments, such as autografts and allografts, have significant limitations, including invasiveness, high costs, and the risk of infections and hematomas at donor or surgical sites [205].

For effective bone regeneration, an ideal graft must be biocompatible, non‐toxic, porous for cell infiltration, mechanically compatible with host bone, and biodegradable at a rate that supports new bone growth [206]. Hydrogels, as candidates in bone tissue engineering, must also exhibit osteoconductive and osteoinductive properties, along with compatibility with host tissues and cells [207]. However, materials used for bone tissue engineering generally need to exhibit both strong mechanical strength and the ability to promote bone growth. While hydrogels are often used as substitutes for bone tissue, they typically have limited mechanical strength, which necessitates improvement [208]. Graphene‐based materials, either alone or combined with bone grafts and scaffolds, can significantly enhance bone regeneration by promoting cell adhesion, proliferation, and differentiation into osteoblasts [209]. Incorporating graphene into hydrogels allows for 3D manipulation, accelerates cell differentiation, and improves osteoconductivityreducing reliance on additional osteogenic agents. Techniques to reduce *π–π* interactions between graphene sheets make it easier for polymers to spread, further advancing bone regeneration [135].

For example, Jiang et al. developed an injectable hydrogel combining alginate‐tyramine, sericin, and GO, cross‐linked enzymatically with HRP/H_2_O_2_. Increasing the GO content reduced the hydrogel's storage modulus, with structural failure occurring beyond a critical strain of 20%. This hydrogel enhanced macrophage migration, promoted M2 macrophage polarization, and supported osteoblastic differentiation of BMSCs. However, GO concentrations above 20 µg/mL induced inflammation and toxicity. Both in vitro and in vivo studies confirmed the hydrogel's ability to reduce inflammation, inhibit fibrous capsule thickening, and promote mineral deposition in rat BMSCs [136]. In a related study, Wang et al. incorporated GO into zwitterionic hydrogels made with maleic anhydride as a crosslinker. This improved the hydrogels' mechanical properties, reduced cytotoxicity, and promoted rBMSC proliferation and osteogenic differentiation [135].

**FIGURE 7:**
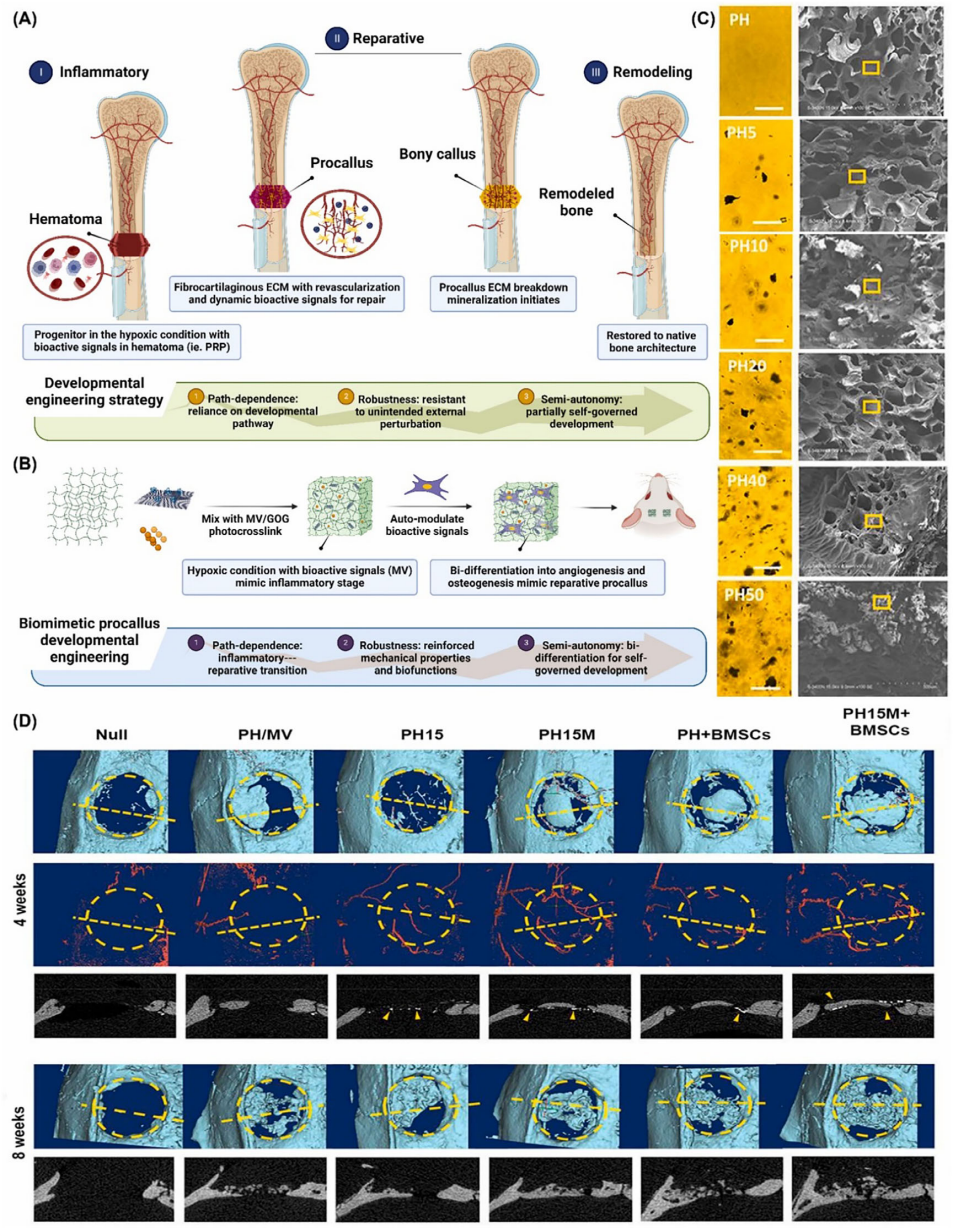
(A) Schematic of bone healing, illustrating path‐dependent and independent features in biomimetic procallus formation. (B) Schematic of biomimetic procallus in developmental engineering, showing how GO/Gel alters the microstructure. (C) SEM and light microscopy images of the composite cross‐section and hydrogel, with a 100‐µm scale bar. (D) Rat calvarial defect repair with biomimetic procallus, evaluated by µCT at 4 and 8 weeks. Yellow dotted lines mark sectional views, and arrowheads indicate contrast‐filled blood vessels with higher radiodensity than bone. Reproduced with permission [137]. Copyright 2020, Elsevier B.V.

Expanding on this, Jiao et al. developed a biomimetic composite combining gelatin and rGO, which exhibited biocompatibility and regulated progenitor cell development. This composite facilitated bone repair by enhancing structural morphogenesis and angiogenic differentiation in BMSCs, as illustrated in Figure 7. The composite's mechanical properties, influenced by its rGO content, supported BMSC adhesion and proliferation, contributing to effective bone regeneration [137].

Cartilage, a specialized connective tissue, is characterized by its smoothness and unique freedom of movement. The main components of cartilage include collagen fibers, glycosaminoglycans, proteoglycans, and a significant amount of water [210]. It provides essential support to the body's structures and is crucial for embryonic growth, as it aids in bone development. Cartilage remains an important part of the skeletal system in adults [211]. The incorporation of graphene into hydrogels for tissue engineering scaffolds capitalizes on its advantageous properties, such as a large surface area, ease of functionalization, exceptional tensile strength, and high electrical conductivity. These attributes help create cell‐compatible environments. They also assist mesenchymal stem cells (MSCs) in differentiating into chondrocyte lineages, such as hyaline cartilage [212, 213]. GO is known for its barrier properties, high surface‐to‐volume ratio, self‐lubrication, and resistance to wear, when incorporated into hydrogel or polymer metrices. It has also been explored for cartilage tissue engineering. For instance, Trucco et al. developed a bilayer hydrogel matrix consisting of gellan gum and polyethylene glycol diacrylate (PEGDA), integrating GO at the top layer. The mechanical features of this design were similar to those of healthy articular cartilage. Tribological tests showed that adding GO enhanced the material's strength and durability. Additionally, biocompatibility tests conducted over six days demonstrated that the bilayer hydrogel system did not harm human chondrocytes, confirming its safety [138].

Expanding on biomimetic scaffold development, Hou et al. developed CHGs using a novel freeze‐thaw and heating process to combine GO‐NSHs, nGO‐APU (modified anionic polyurethane), and PVA. The scaffold's strength was derived from annealed PVA (a‐PVA), and the interwoven nGO‐APU chains improved its water retention and reduced friction. This hydrogel performed well under compressive pressure, similar to the temporomandibular joint (TMJ) disc. When implanted into rabbit TMJs and monitored for 24 weeks, it maintained its shape, protected against cartilage degradation, and mitigated osteoarthritis progression. Finite element analysis [214] further demonstrated its efficiency in stress distribution and energy dissipation under realistic loading conditions [139].

Complementing these efforts, Fu et al. published a study describing a new hydroxyapatite (HA) composite layer capable of self‐cleaning and preventing bacterial growth. This was achieved by vacuum‐infiltrating GO hybrid pseudopolyrotaxane (PPR) supramolecular hydrogels and allowing them to self‐assemble via host‐guest interactions. Infused with vancomycin, these hydrogels exhibited prolonged drug release and a gel‐sol transition in response to shear stress and frictional heat, similar to the expulsion of synovial fluid during joint movement. This system offered exceptional anti‐wear, self‐lubricating capabilities, and antibacterial effects against Staphylococcus aureus. Compared to textured HA coatings, this composite demonstrated significantly enhanced performance in friction reduction and wear resistance [140].

Additionally, Jiang et al. developed a novel hybrid hydrogel system: a glycerol‐modified PVA hydrogel (Pg) reinforced with a 3D‐printed poly(ɛ‐caprolactone) (PCL)‐graphene composite scaffold (PG). Optimization of the hydrogel composition in the hybrid material resulted in high water retention and increased stiffness. The optimized 3D‐printed PCL‐graphene scaffold‐reinforced hybrid hydrogel exhibited mechanical properties (stiffness, toughness, and tribology) comparable to those of natural load‐bearing cartilage [130].

**FIGURE 8:**
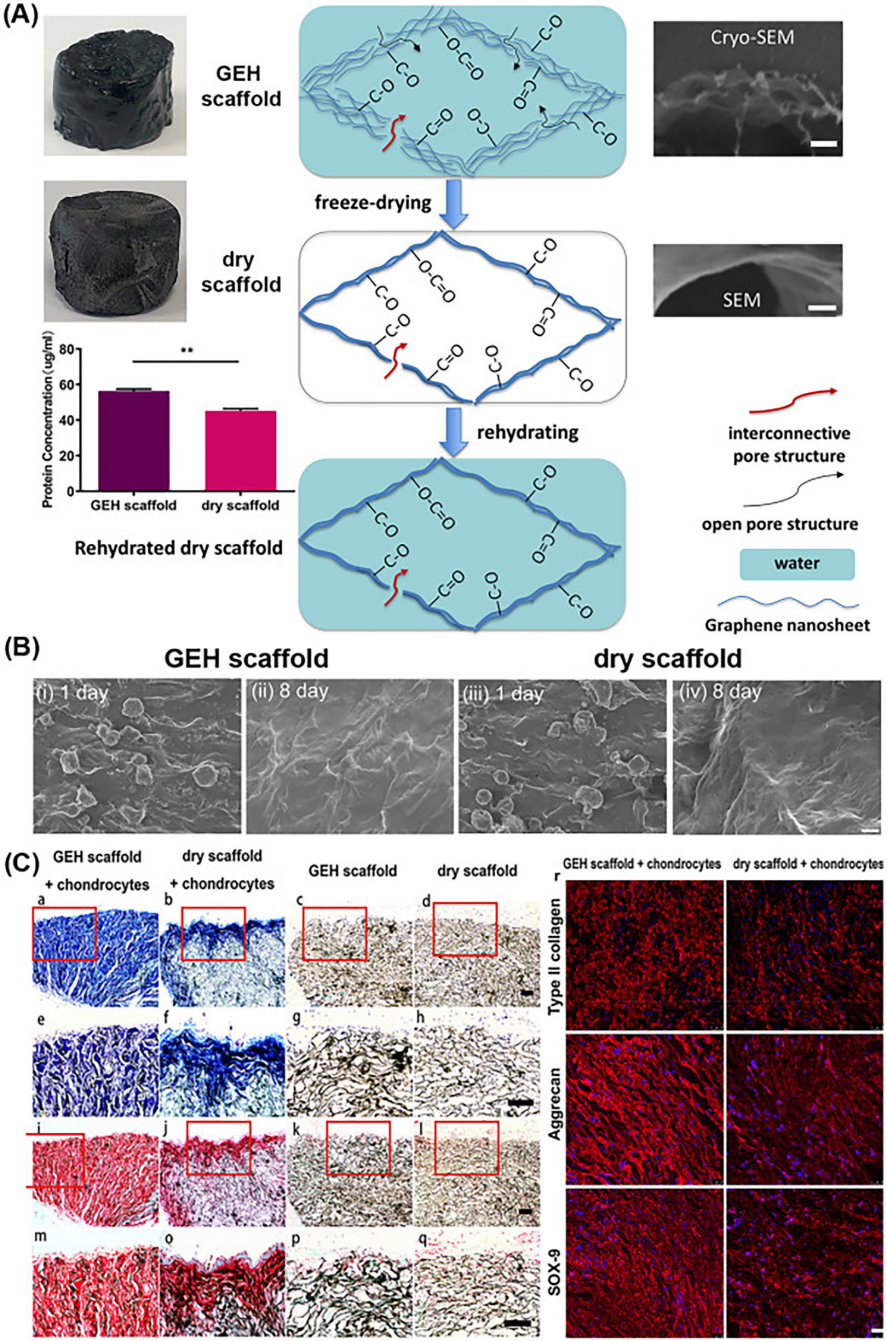
(A) Schematic showing structural features of GEH and dry scaffolds, including interconnected and open porosity, large surface area, and moderate oxygen content. (B) SEM images of scaffold surfaces at days 1 and 8 post‐chondrocyte seeding. (C) Histological staining (Toluidine Blue and Safranin O/Fast Green) of chondrocyte‐seeded and control (cell‐free) scaffolds at 8 weeks post‐implantation. Panels (e–h) show higher magnification of boxed regions (a–d), while (i–q) provide enlarged views of selected areas in (i,l). (r) Immunofluorescence images showing expression of type II collagen, aggrecan, and SOX‐9 in dry and chondrocyte‐seeded GEH scaffolds at 8 weeks. Reproduced with permission [141]. Copyright (2022), American Chemical Society.

Graphene‐based hydrogels have shown promise in enhancing type II collagen production, supporting chondrocyte proliferation, and remodeling the cartilage matrix. Lyu et al. used freeze‐casting techniques with partially reduced GO suspensions to create a graphene elastic hydrogel [215] framework, as shown in Figure 8. This scaffold maintained structural integrity, resisted enzymatic degradation, and quickly regained its original shape after repetitive compression in saline environments. Chondrocytes cultured on the GEH scaffold exhibited elevated production of metalloproteinases and their inhibitors, indicating a favorable environment for cartilage matrix remodeling. Histological analysis revealed that GEH scaffolds, particularly those with porous external structures, significantly promoted cartilage regeneration compared to dehydrated counterparts [141].

#### 5.2.2 Soft Tissue Regeneration

Soft tissue engineering focuses on the repair, regeneration, or replacement of tissues such as skin, muscles, blood vessels, and nerves. These tissues require scaffolds with high elasticity, biocompatibility, and support for vascularization [216]. Graphene‐based CHGs have significantly enhanced the design of hydrogels and scaffolds for soft tissue regeneration.

Human neural tissue is composed of two main components: the peripheral nervous system (PNS), which includes neurons distributed throughout the body, and the central nervous system (CNS), consisting of the brain and spinal cord [217]. These systems work in concert to maintain homeostasis and respond to external stimuli. While minor injuries to the PNS often heal spontaneously, severe damage typically necessitates surgical intervention. In contrast, the CNS exhibits limited regenerative capacity, and functional recovery following injury—particularly in the brain—is exceptionally rare [218].

Hydrogels hold great promise for neural tissue repair, especially when functionalized with chemical agents such as TEMPO or photosensitive molecules. These modified hydrogels adapt to irregularly shaped neural lesions, providing a supportive environment for healing and regeneration [203]. Furthermore, hydrogels can transmit mechanical signals to brain cells, promoting cell proliferation, growth, paracrine signalling, and other processes essential for neural tissue regeneration [219]. In a notable study, Rezaei et al. explored the effects of incorporating GO into collagen (Col)‐based hydrogels to enhance the biological properties of neural stem/precursor cells (NS/PCs). Their results showed that incorporating 1%–1.5% GO significantly improved NS/PC survival, migration, neurite extension, cell spreading, and aggregation—effects that were especially pronounced in Col/GO composite hydrogels. GO enhanced interactions between neural stem cells and the collagen matrix by introducing micro‐ and nanostructures and improving physicochemical cues, thereby facilitating greater cellular engagement. Additionally, GO altered the mechanical flexibility of the hydrogels, further promoting neural stem cell activity [142]. Expanding on electroactive scaffold development, Liu et al. developed an electrically conductive hydrogel by chemically integrating carbon nanotube poly(ethylene glycol) acrylate (CNTpega) and graphene oxide acrylate (GOa) into an oligo(polyethylene glycol fumarate) matrix. GOa was subsequently reduced using L‐ascorbic acid to enhance biocompatibility. This conductive hydrogel markedly promoted the growth, spreading, and neurite outgrowth of PC12 cells [143].

**FIGURE 9:**
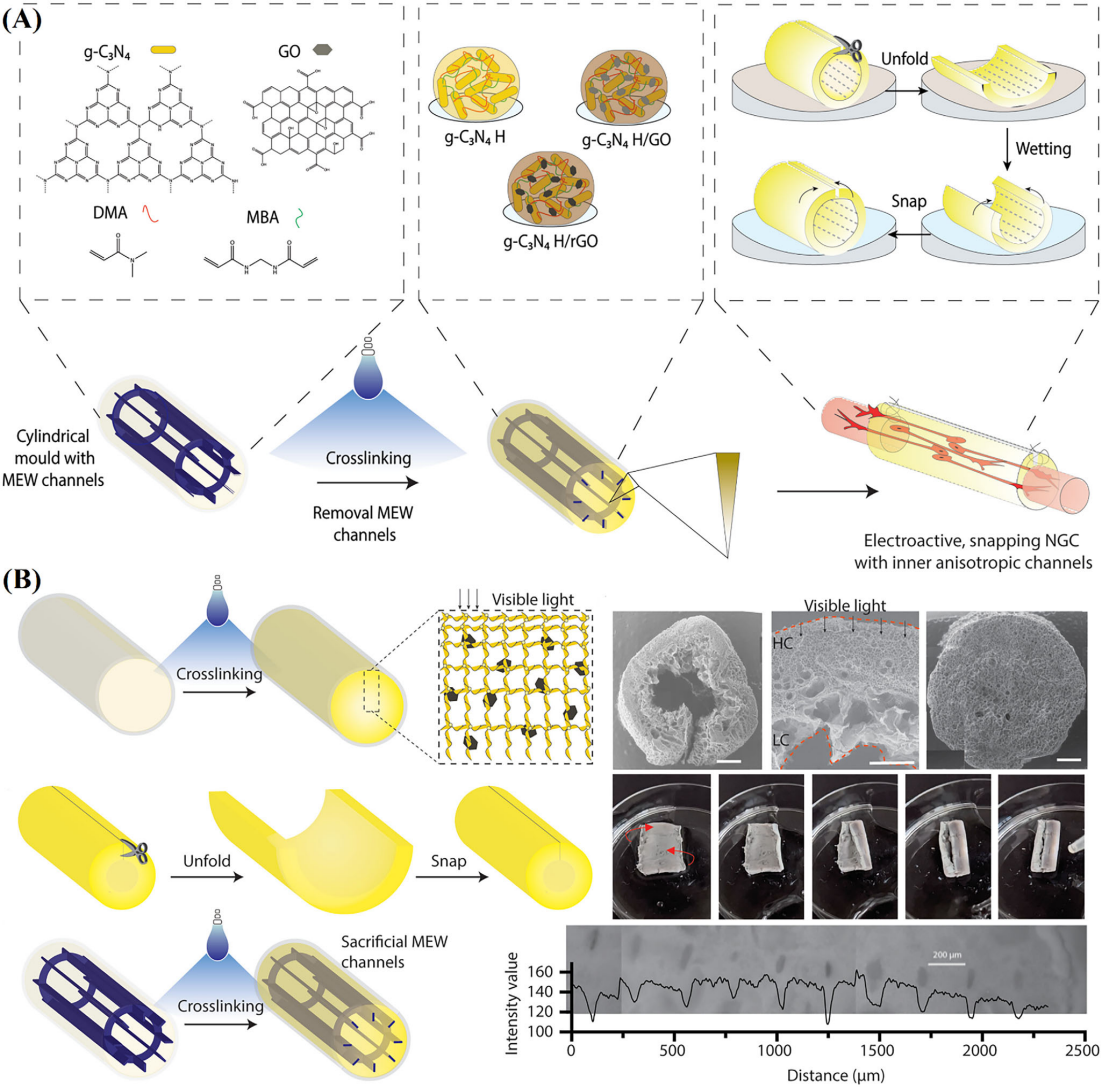
(A) Schematic of a self‐snapping NGC with anisotropic conductivity. The g‐C_3_N_4_ hydrogels were synthesized via blue light‐initiated crosslinking of DMA and MBA. (B) Fabrication of a snapping NGC with built‐in anisotropic sacrificial microchannels. Reproduced with permission [144]. Copyright 2022, Elsevier B.V.

In another advancement, Amagat et al. investigated neural development using a conductive hydrogel composed of graphitic carbon nitride (g‐C_3_N_4_) and rGO, as illustrated in Figure 9. This hydrogel functioned effectively as a nerve guidance conduit (NGC) and demonstrated substantial potential for peripheral nerve repair. Incorporating rGO into the g‐C_3_N_4_ hydrogel matrix increased the surface charge and imparted self‐snapping properties, facilitating surgical implantation. The composite hydrogel exhibited mechanical stiffness well‐suited for peripheral nerve regeneration, while the presence of electroactive rGO significantly enhanced neural differentiation. Notably, the neurite outgrowth of PC12 cells cultured on the g‐C_3_N_4_/H/rGO hydrogel was 47% greater than that observed with the g‐C_3_N_4_/H alone. Furthermore, the integration of sacrificial melt electrowriting (MEW) microchannels enabled directional cell guidance. Overall, the hydrogel supported both neural cell proliferation and differentiation, highlighting its strong potential for therapeutic nerve regeneration [144].

Tendons are strong, flexible connective tissues that link muscles to bones. They are primarily composed of type I collagen fibrils and populated by tenocytes, the specialized cells responsible for maintaining tendon structure [220]. In addition to collagen, tendons also contain proteoglycans, glycoproteins, and elastin, which contribute to their mechanical properties. Tendon injuries can severely compromise their load‐bearing capacity and structural integrity [221]. Despite progress in tissue engineering, fully restoring tendon function remains a significant challenge, as current therapeutic approaches often fail to achieve effective regeneration [222]. To overcome these limitations, functionally engineered hydrogels have been developed by incorporating bioactive agents, pharmaceutical compounds, or reinforcing materials to enhance tendon repair [223, 224].

Among these materials, GO stands out for its potential as a drug delivery vehicle, capable of gradually releasing therapeutic agents and growth factors to support tissue regeneration. For instance, Bao et al. developed a GO‐based system by incorporating GO into platelet‐rich plasma (PRP) gels to enable sustained release of growth factors. PRP was prepared using double centrifugation of rabbit whole blood, followed by the integration of varying GO concentrations into the gels. This system was applied to repair supraspinatus tendon injuries in a rabbit model. The regenerated tissue closely resembled native tendon, and the hydrogel demonstrated excellent biocompatibility. Additionally, it enhanced mechanical properties and supported bone marrow stem cell (BMSC) proliferation, osteogenesis, and chondrogenic differentiation, thereby accelerating tendon healing [145].

In another study, Barzegar et al. improved gel‐based hydrogels for cell growth and differentiation by developing polyglycerol‐functionalized rGO (PG), polyglycerol‐functionalized molybdenum disulfide (PMoS_2_), and PG/PMoS_2_. This combination enhanced the hydrogel's mechanical strength and promoted tendon regeneration, while PG functionalization contributed to reduced inflammation during healing. In vivo studies confirmed that the composite hydrogel improved the biomechanical performance, functional index, and adhesion score of healing Achilles tendons [146]. Yoon et al. also explored the benefits of incorporating a small amount of GO into sodium alginate (NAlg) scaffolds, enhancing their mechanical properties without compromising cell viability. This modification supported the repair of rotator cuff injuries [147].

Overall, recent studies demonstrate that 2D graphene‐integrated CHGs exhibit significant potential for tissue engineering applications by providing biomimetic environments that enhance cellular interactions and structural support. Several graphene‐functionalized hydrogel systems, including GelMA‐, PEG‐, and polysaccharide‐based matrices reinforced with GO or rGO, have been reported to enhance cell adhesion, proliferation, and lineage‐specific differentiation, while also improving structural stability and functional performance. These advances highlight the versatility of graphene derivatives in supporting bone, cartilage, neural, tendon, and soft tissue regeneration in preclinical models. Nevertheless, challenges related to long‐term biocompatibility, controlled biodegradation, and scalable fabrication remain, underscoring the need for continued optimization and systematic in vivo evaluation to facilitate future translational and clinical applications.

### 5.3 Photothermal Therapy/ Cancer Therapy

PTT is an alternative cancer treatment that destroys tumor cells using heat [225]. The temperature rise caused by NIR light leads to localized photocoagulation [226]. Hydrogels are extensively used in PTT due to their unique properties. However, their application can be limited by insufficient mechanical strength in conventional hydrogel systems [227]. Adding additional materials to hydrogels can help address this issue. These materials enhance the structural stiffness and better mimic the extracellular matrix (ECM) [183]. Graphene‐based CHGs generate heat under NIR irradiation through the efficient PT conversion properties of graphene and its derivatives, such as GO and reduced rGO [43]. These 2DMats have delocalized π‐electron systems that strongly absorb NIR light (typically in the 808–1064 nm range), leading to electronic excitation [43]. The absorbed energy is then non‐radiatively dissipated as heat via electron–phonon and phonon–phonon interactions [228]. Embedded in a hydrogel matrix, these 2D materials serve as localized heat sources, while the polymer network helps localize and moderate heat distribution. The resulting hyperthermia (> 42°C) can disrupt cellular homeostasis by inducing mitochondrial dysfunction, reactive oxygen species (ROS) generation, and activation of apoptosis related pathways [229, 230]. Additionally, thermal stress upregulates heat‐shock proteins (e.g., HSP70, HSP90), compromises membrane integrity, and may alter the tumor microenvironment, potentially enhancing vascular permeability and immune cell infiltration [231, 232]. These combined effects enable precise, localized tumor ablation while minimizing off‐target tissue damage, making graphene‐based hydrogels highly promising platforms for non‐invasive, multimodal cancer therapy [194].

**FIGURE 10:**
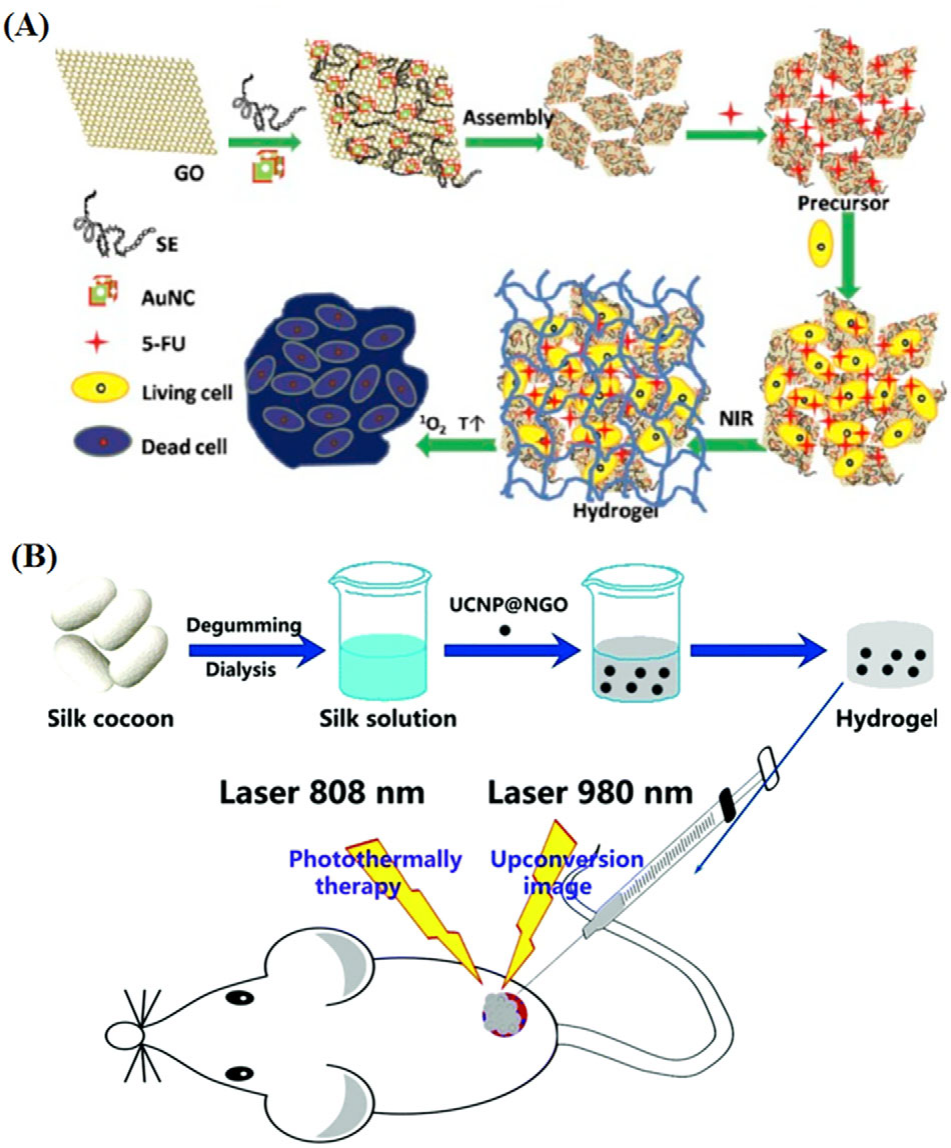
(A) Synthetic process and antitumor mechanism of the 5‐FU‐loaded composite hydrogel. Reproduced with permission [148]. Copyright 2016, Elsevier B.V. (B) Synergistic upconversion luminescence imaging and PTT with an injectable SF/UCNP@NGO hybrid hydrogel. Reproduced with permission [149]. Copyright 2019, Royal Society of Chemistry.

Building upon this conceptual foundation, Chang et al. developed a composite hydrogel for targeted and multimodal drug delivery. This system consisted of spinach extract (SE), rGO, and gold nanocages (AuNCs), as shown in Figure 10A. SE played multiple roles: it facilitated hydrogel formation, enhanced biocompatibility, and acted as a natural photosensitizer, enabling tumor cell destruction under 660 nm laser irradiation. AuNCs exhibited strong PT properties and boosted the production of cytotoxic singlet oxygen (^1^O_2_). The composite hydrogel shell improved tumor treatment by enabling localized drug delivery, PDT/PTT compatibility, and controlled 5‐FU release. This synergy enhanced multimodal therapy. When HeLa cells were incubated with the 5‐FU‐loaded hydrogel and exposed to NIR irradiation (10 min), cell survival sharply dropped to 1.2%, demonstrating a significant anticancer effect. These findings highlight the hydrogel's potential for localized, NIR‐responsive combination therapy, integrating PTT, PDT, and chemotherapy for enhanced antitumor effects [148].

Building on this concept of multifunctional platforms for cancer treatment, He et al. fabricated another hybrid hydrogel system using silk fibroin [233] nanofibers combined with upconversion nanoparticles and nano‐graphene oxide (UCNP@NGO) hybrid hydrogels (SF/UCNP@NGO), as shown in Figure 10B. The strong PT synergistic effect observed in SF/UCNP@NGO hydrogels under NIR laser irradiation was primarily due to NGO's high PT conversion efficiency. Among various therapies, PTT showed the most effective tumor growth suppression in mouse models. Additionally, the combination of upconversion luminescence (UCL) imaging and PTT helped reduce dose‐limiting toxicity and minimize tissue damage caused by overheating, thereby improving therapeutic efficiency. Due to these advantages, the SF/UCNP@NGO hydrogel system shows great potential for multifunctional anti‐tumor therapy and future clinical applications [149].

**FIGURE 11:**
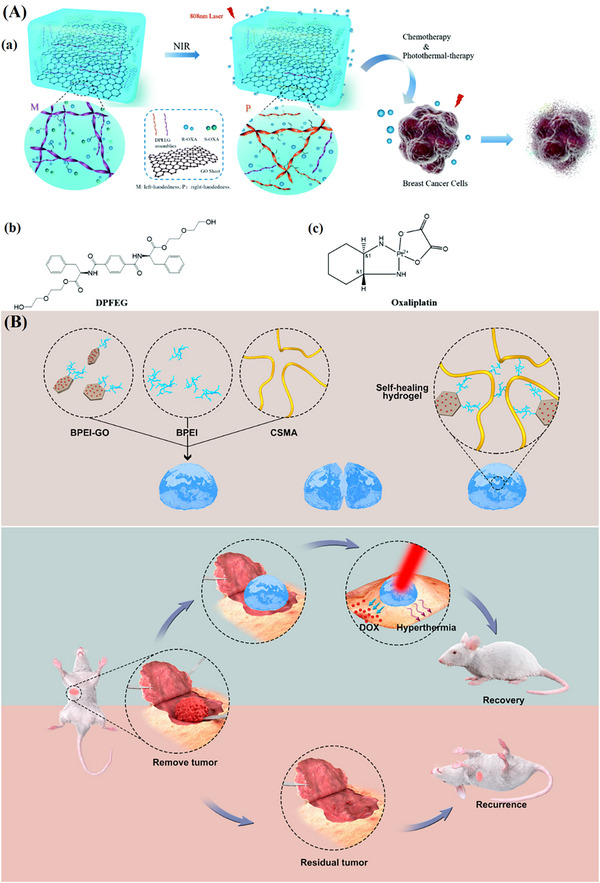
(A) (a) Phenylalanine‐based hydrogel with GO for selective chiral anticancer drug delivery under IR irradiation. Structures of (b) DPFEG and (c) oxaliplatin. Reproduced with permission [150]. Copyright 2022, Royal Society of Chemistry. (B) Diagrammatic representation of CSMA/BPEI/BPEI hydrogel for preventing breast cancer recurrence. Reproduced with permission [151]. Copyright 2019, American Chemical Society.

Expanding on this approach, Wang et al. developed a chiral hydrogel system by co‐assembling a D‐phenylalanine derivative gelator (DPFEG) with GO, as shown in Figure 11A. The release of the chiral anticancer drug oxaliplatin was precisely controlled using NIR light to manipulate chirality. Without NIR light, the drug remained trapped in the system for up to 7 h. However, NIR irradiation triggered its release. Additionally, GO contributed to PTT due to its efficient light‐to‐heat conversion. As a result, DPFEG‐GO significantly inhibited the growth of T47D breast cancer cells. This advanced system offers a simple and effective method for chiral drug absorption and controlled release. It also enables a combined chemotherapy and PTT approach for cancer treatment [150].

In a similar vein, Li et al. developed a self‐healing hydrogel using graphene nanoparticles to treat breast cancer recurrence after surgery. They first synthesized the hydrogel using a facile method based on Schiff base linkages. The hydrogel consisted of chondroitin sulfate multi‐aldehyde (CSMA), branched polyethylenimine (BPEI), and BPEI‐conjugated graphene (BPEI‐GO), as shown in Figure 11B. BPEI‐GO, integrated into the hydrogel network, underwent Schiff base reactions. This process stabilized the structure, allowed sustained drug delivery, and enabled a NIR‐induced PT effect. The hydrogel demonstrated nearly complete self‐healing efficiency (approaching100% under test conditions) and improved mechanical strength (7000 Pa). In vitro breast cancer studies showed that the hydrogel significantly enhanced cell death through a combined chemo‐PTT approach. In a breast cancer recurrence prevention mouse model, the combination of DOX and PTT within CSMA/BPEI/BPEI‐GO hydrogels reduced tumor recurrence to 33.3%. This was significantly lower than other groups (66.7%–100%). These findings highlight the promising potential of CSMA/BPEI/BPEI‐GO hydrogels for preventing breast cancer recurrence after surgery [151].

**FIGURE 12:**
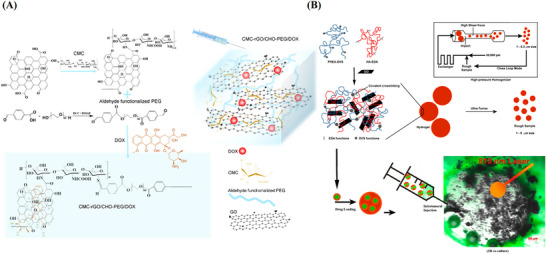
(A) CMC‐rGO/CHO‐PEG hydrogel fabrication, drug loading, and DOX release curves. Reproduced with permission [152]. Copyright 2018, Elsevier B.V. (B) Schematic of NG synthesis, drug loading, intratumoral injection, and PT effect against HCT 116 cells.Reproduced with permission [153]. Copyright 2017, American Chemical Society.

Expanding on this concept, Liu et al. developed a novel PTT agent using a carboxymethyl chitosan‐functionalized reduced graphene oxide (CMC‐rGO) complex combined with a dialdehyde‐functionalized poly(ethylene glycol) (CHO‐PEG) hydrogel (CMC‐rGO/CHO‐PEG). This hydrogel demonstrated strong NIR absorbance, pH responsiveness, and controlled DOX release. By modifying the molecular weight and functional groups of PEG precursors, researchers adjusted the mechanical properties and scaffold architecture of CHO‐PEG hydrogels. In this study, CMC‐rGO nanosheets (NSHs) were synthesized through amidation, significantly enhancing chemo‐photothermal efficiency and biocompatibility. When exposed to NIR laser irradiation, CMC‐rGO, non‐covalently functionalized with CHO‐PEG hydrogels, acted as a biocompatible and effective chemo‐photothermal agent (Figure 12A). This hydrogel uniquely combined biodegradability, synergistic therapy, and controlled drug release. The synthesis method offered several advantages: it was simple, eco‐friendly, efficient, and cost‐effective. The drug release mechanism relied on physical diffusion and the breakdown of Schiff‐base bonds. These findings highlight the potential of NIR‐absorbing PTT agents for cancer treatment, paving the way for further studies on novel 3D NCs for biomedical applications [152].

Complementing these efforts, Fiorica et al. developed hyaluronic acid (HA)/polyaspartamide nanogels (NG) as a potential treatment for colorectal carcinoma. The nanogels were synthesized using hyaluronic‐((2‐aminoethyl)‐carbamate) acid (HA‐EDA) and α,β‐poly(N‐2‐hydroxyethyl)‐D,L‐aspartamide‐divinyl sulfone (PHEA‐DVS), as shown in Figure 12B. GO, with its large aromatic surface area, efficiently incorporated a high amount of irinotecan (IT) (33.0% w/w), exhibiting strong hyperthermic effects under NIR laser irradiation. The release of the antitumor drug was influenced by both external pH and NIR irradiation. Erythrolysis studies confirmed the safety of the nanogels in contact with blood cells, suggesting their potential for intravenous administration. In vitro experiments showed that hyperthermia significantly enhanced the cytotoxicity of the antitumor drug against HCT 116 cells. However, 3D coculture studies indicated that direct tumor ablation might be achievable through local NG injection [153].

**FIGURE 13:**
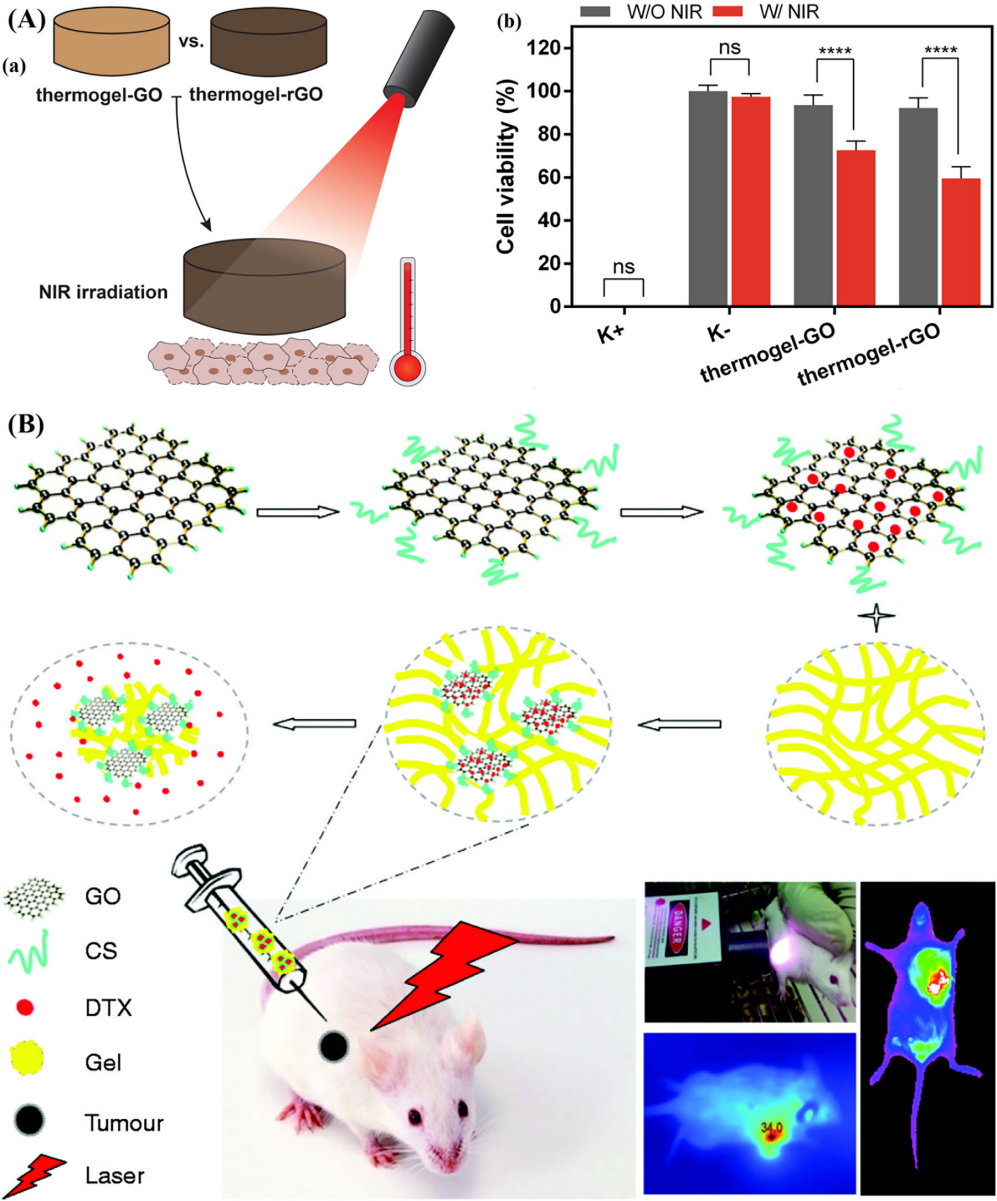
(A) PTT mediated by thermogel‐GO and thermogel‐rGO (a). Effect on MCF‐7 cells (10 µg/mL GO or rGO) with or without NIR irradiation (b). Data: mean ± SD, *n* = 5 (*p* < 0.0001, ns = non‐significant). Reproduced with permission [154]. Copyright 2020. Elsevier B.V. (B) Illustration of DTX–GO/CS gel for chemo‐PTT. Reproduced with permission [155]. Copyright 2016, SAGE Publications.

Pushing this direction further, Sousa et al. developed a hydrogel system for targeted tumor delivery of GO/rGO using injectable, thermo‐responsive chitosan‐agarose hydrogels. These hydrogels, containing GO or rGO, served as 3D matrices and exhibited good injectability, gelation kinetics, physicochemical properties, and cytocompatibility. Upon NIR light irradiation, thermogel‐rGO showed a 3.8‐fold higher temperature increase than thermogel‐GO. This heat effect reduced breast cancer cell viability to 60%. A chemo‐photothermal effect was achieved by optimizing the DOX: Ibuprofen ratio in the thermogel‐rGO formulation, further lowering cancer cell viability to 34%. Additionally, NIR laser irradiation enhanced the antibacterial properties of the hydrogels, reducing infection risks at the application site. As shown in Figure 13A, the injectable, self‐assembling thermogel‐rGO hydrogel presents a promising strategy for breast cancer treatment by combining chemo‐PTT with NIR‐enhanced therapy [154].

Building on this strategy, Zhang et al. developed a thermosensitive, GO‐based hydrogel loaded with DTX for intratumoral delivery. This system aimed to enhance treatment efficacy while reducing systemic toxicity. The pH‐sensitive DTX–GO/CS gel exhibited a significantly slower DTX release rate compared to stable GO/CS in physiological solution. In vitro, DTX–GO/CS showed greater antitumor efficacy in MCF‐7 cells than free DTX. In a mouse tumor model, intratumoral injection of the DTX–GO/CS gel resulted in higher and more sustained drug concentrations within tumor tissues, with no significant toxicity to normal organs. Additionally, 808 nm NIR laser irradiation significantly enhanced tumor growth inhibition in both in vitro and in vivo models. As illustrated in Figure 13B, these findings highlight the potential of this hydrogel system for cancer chemo‐PTT therapy [155].

**FIGURE 14:**
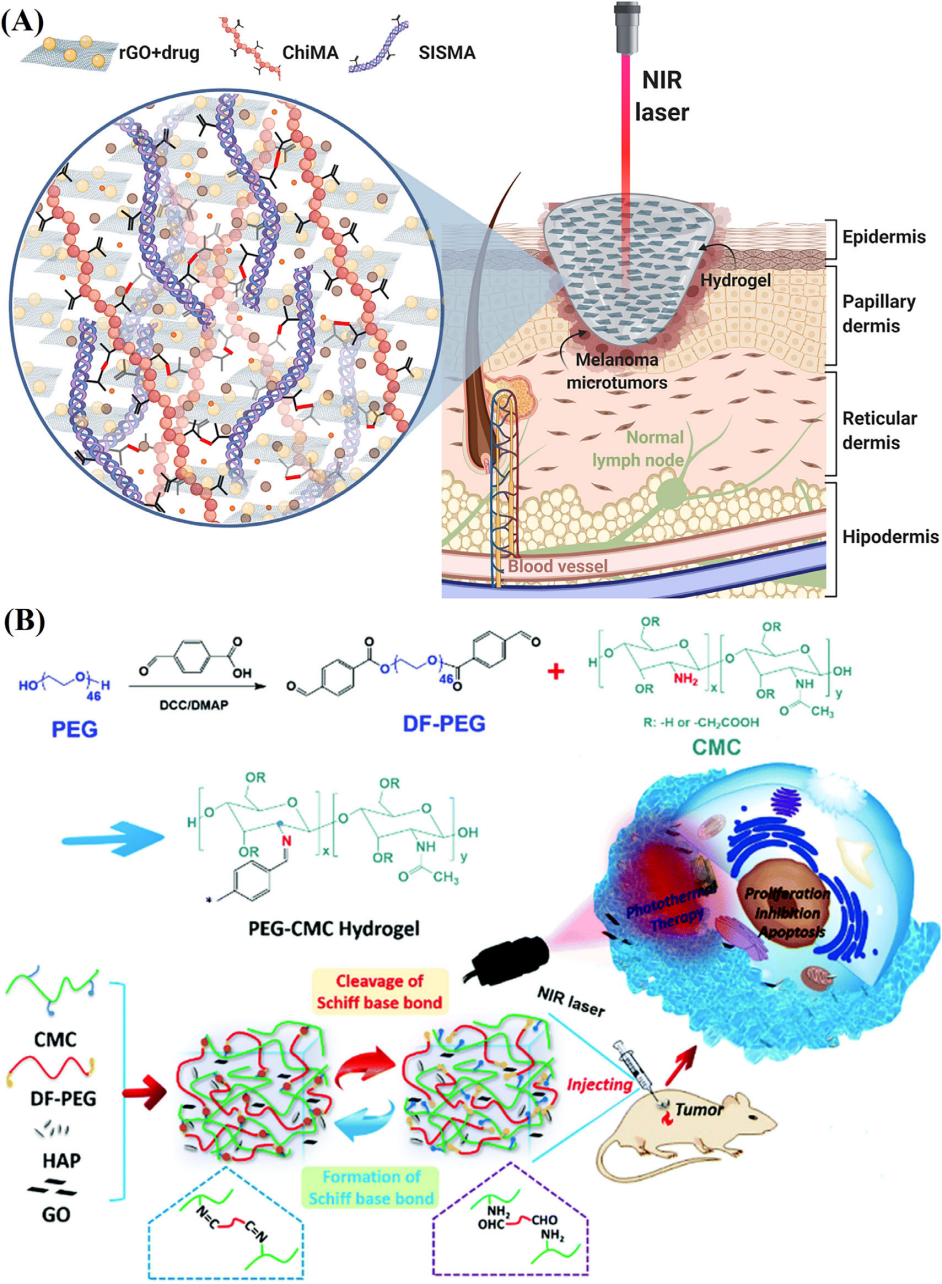
(A) Schematic of the SISMA/ChiMA/rGO composite hydrogel for chemo/PTT targeting microtumor residues post‐surgical resection of cutaneous melanoma. Reproduced with permission [156]. Copyright 2022, Frontiers Media S.A. (B) Schematic of the injectable, self‐healing PEG–CMC/HAP/GO NC hydrogel, synthesized via DF‐PEG reaction, combining synergistic inhibition therapy and PTT. Reproduced with permission [157]. Copyright 2021, Royal Society of Chemistry.

Similarly, Valenzuela et al. developed a stimuli‐responsive hydrogel for eliminating microtumor remnants after melanoma surgery. Their system combined NIR light‐triggered PTT with thermally induced release of DOX, as shown in Figure 14A. The hydrogel was synthesized using chitosan methacrylate (ChiMA), porcine small intestine submucosa methacrylate (SISMA), and rGO‐DOX. This formulation resulted in a photo‐responsive SISMA/ChiMA/rGO‐DOX composite hydrogel. The NC hydrogel enabled in situ deposition and crosslinking. Additionally, incorporating ascorbic acid in its hydrated form facilitated the self‐reduction of GO at body temperature. Acting as a biomimetic adhesive, the hydrogel exhibited potent PT properties, making it suitable for treating tumor sites post‐surgery. Moreover, modified GO functioned as a temporary carrier, binding and deactivating DOX within a biomimetic wound‐healing matrix. This system created a multifunctional platform for stimulus‐triggered drug release and post‐treatment healing [156].

In pursuit of non‐chemotherapy cancer therapies with reduced systemic toxicity, Qi et al. proposed a novel Schiff base cross‐linked hydrogel system using aldehyde‐functionalized PEG and CMC, as depicted in Figure 14B. This hydrogel serves as a delivery system for GO and needle‐shaped nano‐hydroxyapatite (HAP, a tumor inhibitor). The PEG–CMC/HAP/GO NC hydrogel system integrates tumor proliferation inhibition therapy with PTT, enhancing treatment precision while minimizing chemotherapy‐related side effects. Its mechanical properties—including a porous structure, good injectability, and self‐healing ability—meet the necessary biomedical requirements. In vitro studies confirmed GO's phototoxicity to tumor cells, HAP's anti‐proliferative effects, and the hydrogel's retention at the tumor site with no detectable GO/HAP transfer to or observable damage inhealthy tissue. An in vivo study on breast cancer, using a mouse model, involved intratumoral injections of the PEG–CMC/HAP/GO NC hydrogel. The incorporation of GO and HAP into the self‐healing hydrogel significantly suppressed tumor cell proliferation and demonstrated synergistic PTT effects, highlighting a promising approach for tumor treatment [157].

To conclude, the incorporation of graphene‐based NC hydrogels has significantly enhanced the effectiveness of PTT for cancer treatment. Different graphene‐based hydrogels have performed well to treat the different cancer types. Among the most promising advancements, the SE‐rGO‐AuNC hydrogel has demonstrated exceptional multimodal capabilities, achieving substantial HeLa cell destruction under NIR irradiation. Similarly, the SF/UCNP@NGO hydrogel has combined PTT with upconversion luminescence imaging, minimizing tissue damage while enabling precise tumor ablation. The CSMA/BPEI/BPEI‐GO hydrogel has successfully reduced breast cancer recurrence post‐surgery, highlighting its potential in postoperative cancer management. Moreover, the DPFEG‐GO system has enabled controlled, NIR‐triggered release of chiral drugs, optimizing chemotherapy and PTT synergy. These innovative hydrogels not only address conventional limitations, such as poor mechanical strength and inefficient drug release, but also introduce self‐healing, biodegradable, and stimuli‐responsive features. As a result, graphene‐based hydrogels continue to pave the way for highly effective, localized, and minimally invasive preclinical cancer treatments with enhanced therapeutic precision. Despite these advancements, challenges such as long‐term biocompatibility and large‐scale clinical translation remain. Future efforts should refine hydrogel formulations to enhance biodegradability, stability, and targeted delivery.

## 6 ML‐Assisted Design and Applications of Graphene‐Based CHGs

The integration of artificial intelligence (AI), especially ML, has significantly advanced the design and optimization of graphene‐based CHGs. It helps address key challenges in predicting material performance, refining structures, and enhancing functional adaptability [234, 235]. Extensive experimental studies have highlighted the unique properties of graphene‐based hydrogels. However, a major challenge remains—establishing robust and quantitative links between synthesis parameters and final material properties [71]. Traditional trial‐and‐error methods are slow, resource‐intensive, and ineffective at capturing complex nonlinear relationships [236]. These relationships exist between hydrogel composition, crosslinking mechanisms, and biomedical performance. This section explores how ML‐driven approaches overcome these challenges. It examines how predictive modeling, inverse design, and real‐time adaptation improve the development of graphene‐polymeric hydrogels for biomedical applications.

ML‐driven approaches, encompassing both supervised and unsupervised learning techniques, leverage complex linear and nonlinear correlations among the structural, mechanical, and physicochemical properties of graphene‐based CHGs [237]. These computational models facilitate predictive analytics, enabling the systematic evaluation of crosslinking mechanisms, rheological behaviors, and functional performance characteristics of polymeric composite hydrogels [238, 239]. The ML modeling pipeline involves data preprocessing, feature extraction, algorithm selection, and iterative validation to enhance predictive accuracy, thereby accelerating the rational design and performance optimization of NC hydrogels for biomedical applications [240, 241].

Graphene's unique physicochemical properties have positioned it as a cornerstone material in AI‐assisted biomedical engineering [242]. However, the real challenge lies in optimizing these properties for specific applications, such as biosensing, drug delivery, and tissue engineering [243]. ML algorithms facilitate this optimization by refining signal processing in graphene‐based biosensors, improving sensitivity, and enhancing real‐time adaptability in wearable devices [244]. In particular, ML‐powered inverse design methodologies—where optimal material properties are defined first, followed by computationally guided synthesis—have been instrumental in developing hydrogels with tunable mechanical and bio‐responsive properties. Such approaches accelerate the discovery of high‐performance materials tailored to medical diagnostics and regenerative medicine [245, 246].

The integration of ML with GO‐based hydrogels further expands their biomedical potential, particularly in cancer diagnostics, targeted drug delivery, and regenerative medicine. AI‐driven predictive modeling enhances the accuracy of GO‐based biosensors, enabling real‐time interpretation of biochemical signals for precision therapeutics. Notably, studies have demonstrated that GO nanosheets exhibit size‐dependent cellular uptake, influencing their absorption efficiency and cytotoxicity. ML frameworks can systematically analyze these dependencies, guiding the rational design of biocompatible graphene NCs with optimized pharmacokinetics and therapeutic efficacy [124].

In the realm of graphene‐based CHGs, ML has significantly advanced the design and optimization of biomedical applications. For instance, in biosensor development, the integration of graphene with plasmonic materials like gold and silver has enabled enhanced sensitivity for detecting hemoglobin levels. ML algorithms, such as Gradient Boosting, have played a pivotal role in predicting sensor behavior, optimizing design parameters, and reducing computational complexity. Results from the Gradient Boosting Algorithm (GBoost) indicate a perfect predictive accuracy (R^2^ = 1.0) for various configurations, drastically improving simulation efficiency by reducing resource consumption by 85%. These AI‐powered optimizations enhance sensor performance metrics, such as sensitivity (267 GHzRIU−1) and resolution (0.094), making the proposed biosensor a promising tool for real‐time health diagnostics, including anemia detection [247]. This combination of graphene with AI in the design of advanced biosensors exemplifies the transformative potential of ML‐assisted applications in improving precision and efficiency in medical technology.

Graphene‐based materials, particularly rGO‐enhanced hydrogels, have also demonstrated significant promise in both biomedical and AI applications, offering unique advantages in sensor development and wearable technologies. For instance, Li et al., who developed a gesture recognition system utilizing rGO‐based hydrogel strain sensors for rehabilitation training, these materials enhance the functionality and performance of wearable devices designed for healthcare applications [248]. The integration of ML with rGO‐enhanced hydrogels allows for accurate, real‐time monitoring of human joint movements, such as finger flexion, with exceptional sensitivity (gauge factor = 6.13) and fast response times (40.5 ms). This system was able to achieve 100% recognition accuracy across nine distinct hand gestures, highlighting the potential for graphene‐based hydrogels in applications like physical rehabilitation, human‐machine interfaces, and motion sensing. The tunable mechanical and electrical properties of these hydrogels, including high stretchability, biocompatibility, and responsiveness, make them ideal candidates for the next generation of wearable health‐monitoring devices, where they can facilitate gesture‐based rehabilitation, assistive technologies, and personalized patient care. Such advancements underscore the critical role of graphene in enhancing both biomedical and AI‐driven systems.

**FIGURE 15:**
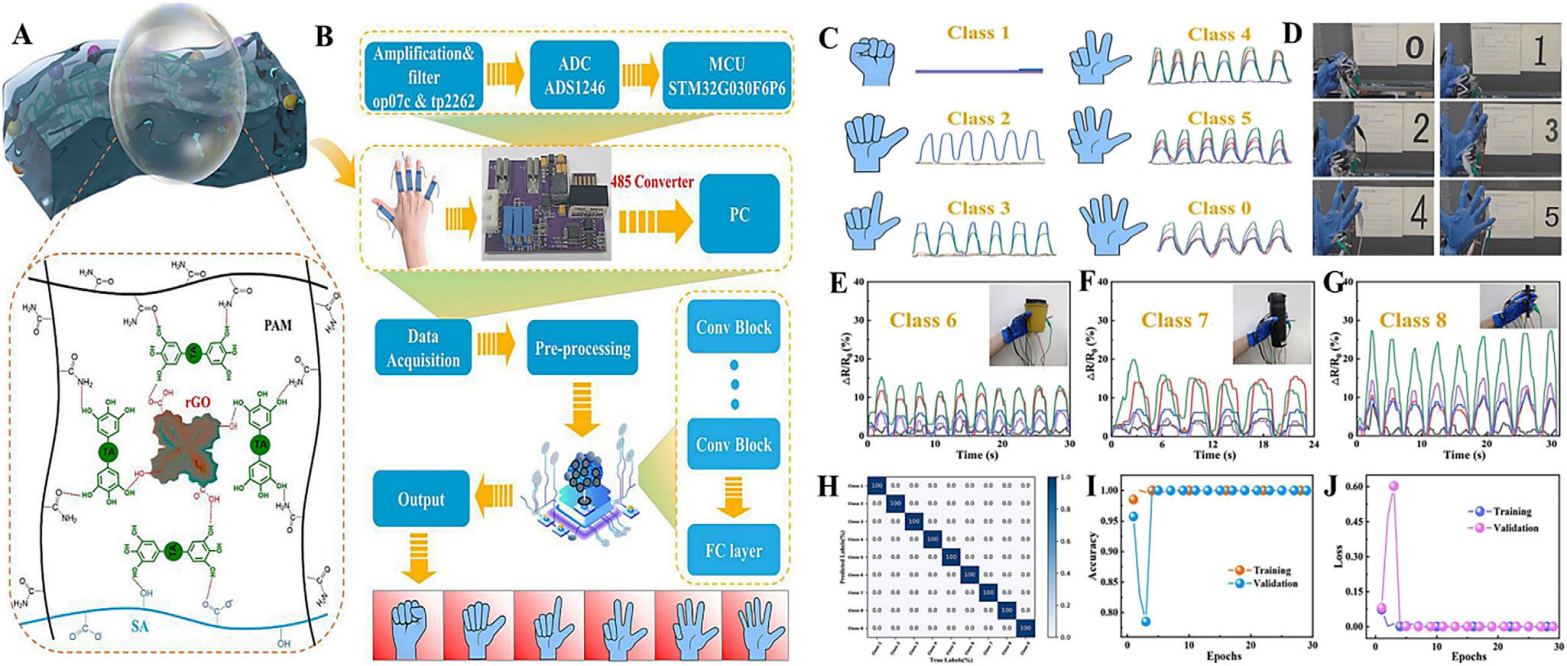
Wearable sensors for human–machine interaction (HMI). (A) Fabrication schematic of PSTG hydrogel. (B) Gesture recognition system flowchart with interactive demonstration. (C) Response curves for distinct five‐finger gestures. (D) Real‐time scene recognition. (E–G) Resistance changes for varying degrees of finger bending. (H) Confusion matrix showing recognition accuracy. (I, J) Gesture recognition accuracy and loss rate tracking. Reproduced with permission [248]. Copyright 2025, American Chemical Society.

Figure 15 illustrates the integration of rGO‐enhanced hydrogels with AI‐driven gesture recognition for biomedical applications. Figure 15A presents the molecular structure of the rGO‐based hydrogel, composed of rGO, polyacrylamide [80], and sodium alginate (SA), which provides high conductivity, flexibility, and biocompatibility, making it well‐suited for wearable strain sensors. Figure 15B outlines the data acquisition and processing framework, where signals from hydrogel strain sensors worn on the fingers are amplified, digitized (ADC ADS1246), and processed using an MCU (STM32G030F6P6) before being transmitted to a computer. These signals undergo preprocessing and feature extraction before being analyzed by a convolutional neural network (CNN) to classify hand gestures. Figure 15C showcases the classification of nine distinct hand gestures based on signal variations corresponding to different finger movements, highlighting the hydrogel sensor's responsiveness and sensitivity. Figure 15D demonstrates real‐time recognition and visualization of hand gestures, reinforcing the system's ability to provide immediate feedback for applications such as rehabilitation training and human‐machine interaction. Figures 15E–G depict the dynamic response of the hydrogel sensors, illustrating the relative resistance change (ΔR/R_0_) for different hand movements (Classes 6–8). The system effectively captures fine motor movements with high precision, validating its real‐time monitoring capability. Figure 15H presents a confusion matrix confirming 100% classification accuracy across all gesture types, further emphasizing the robustness of the AI‐enhanced hydrogel system. Figure 15I,J displays the training and validation accuracy/loss curves, demonstrating the ML model's ability to learn gesture patterns efficiently while minimizing overfitting. These results validate the effectiveness of rGO‐enhanced hydrogels in facilitating highly accurate and responsive gesture recognition, underscoring their potential for advanced wearable technologies in rehabilitation, assistive systems, and human‐machine interfaces.

Graphene‐based CHGs have emerged as a transformative material in biomedical and AI‐driven applications, offering high flexibility, enhanced electrical properties, and compatibility with self‐powered sensing systems. Recent advancements in hydrogel‐based, such as those by Luu et al., who proposed triboelectric nanogenerators (TENGs), demonstrate their potential for real‐time human motion recognition, leveraging graphene nanoplatelets (GNP) to enhance electrical conductivity and mechanical durability [111]. A study on water‐assisted recovered hydrogels incorporated GNP within a cross‐linked polyethylene oxide‐PVA matrix, optimizing charge transport and ensuring stable electrical output after repeated hydration cycles. These hydrogels exhibited superior performance, generating ∼594 V, 40 µA, and 32 nC after multiple recovery cycles, withstanding over 16 000 deformation cycles, and achieving stretchability up to 541%. The tunable electrical properties of GNP‐integrated hydrogels enabled multi‐modal sensing capabilities, allowing the differentiation of motion signals with minimalbrk wiring.

**FIGURE 16:**
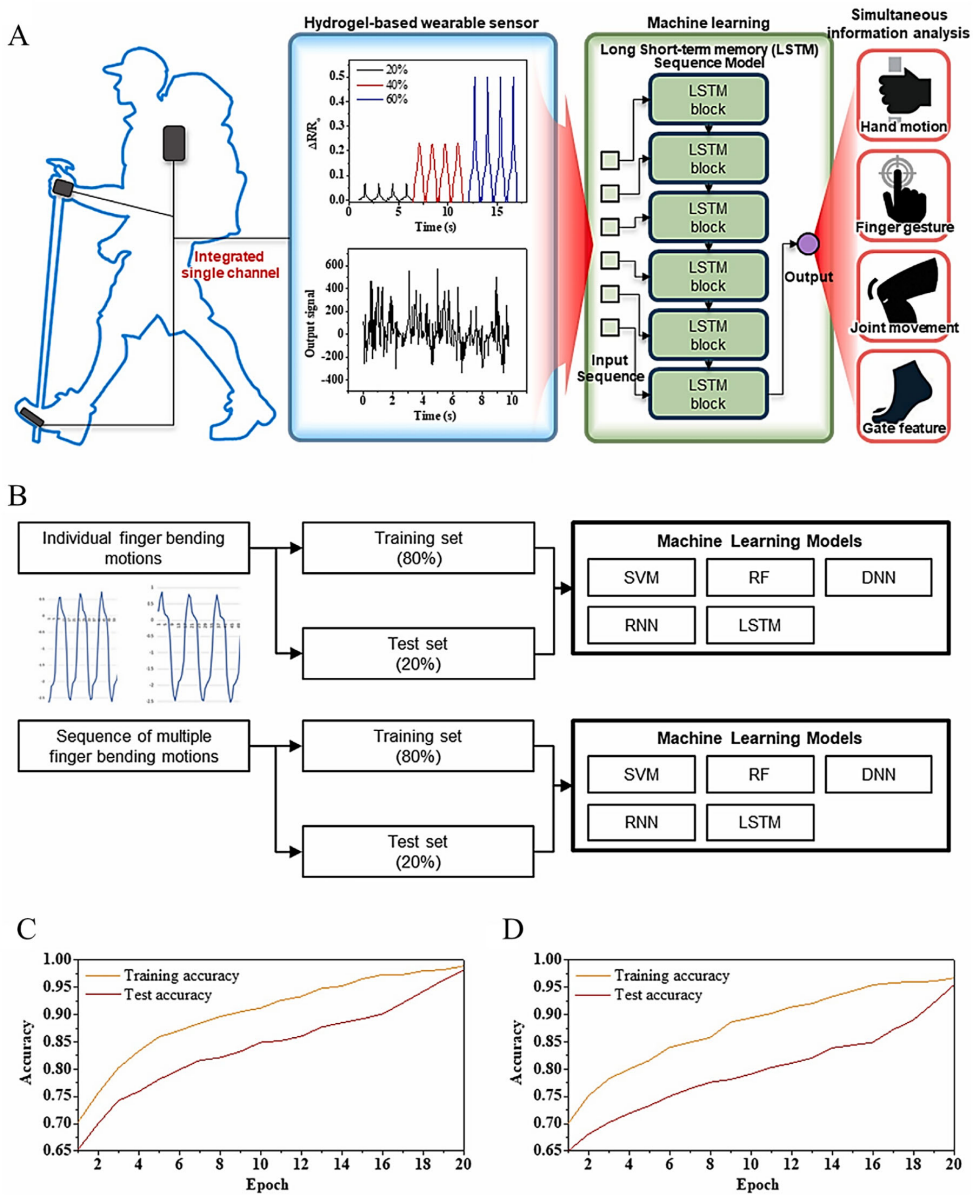
ML‐enabled single‐channel G‐hydrogel sensor system. (A) Overview of human motion detection using G‐TENG and ML. (B) ML workflow using finger bending data. (C,D) Training and test accuracy for single and multi‐finger motion sequences. Adapted with permission [111]. Copyright 2025, Elsevier B.V.

To maximize the efficiency of human motion recognition, recent studies have leveraged ML models trained on finger‐bending motion data extracted from the hydrogel‐based sensors. Figure 16A illustrates the integration of the hydrogel‐based wearable sensor, which captures dynamic resistance changes during motion and processes the data through an LSTM‐based ML model to classify various motion patterns such as hand gestures, joint movement, and gait features.

A comparative evaluation of five ML models—Support Vector Machine (SVM), Random Forest [189], Deep Neural Networks (DNN), Recurrent Neural Networks (RNN), and Long Short‐Term Memory (LSTM) was performed using 5‐fold cross‐validation. The validation accuracies obtained were SVM: 0.925, RF: 0.891, DNN: 0.933, RNN: 0.911, and LSTM: 0.966. As shown in Figure 16B, the dataset was split into training (80%) and testing (20%) sets for both individual and sequential finger‐bending motions. Among the models tested, the LSTM model demonstrated the highest validation accuracy, showcasing its superior ability to capture temporal dependencies within the motion data. Consequently, LSTM was selected as the optimal model for final testing.

As further illustrated in Figure 16C,D, the training and test accuracy trends across epochs highlight the robustness of the proposed ML framework. For individual finger‐bending motions, utilizing resistance measurements alone achieved an accuracy of 0.972, whereas combining resistance and voltage measurements further improved the accuracy to 0.981. Similarly, for sequences of multiple finger‐bending motions, the accuracy improved from 0.922 (resistance only) to 0.954 (combined resistance and voltage). These findings underscore the potential of graphene‐integrated hydrogel sensors, combined with AI‐driven models, for next‐generation wearable electronics, human‐machine interfaces, and real‐time biosensing applications.

The integration of ML in graphene‐polymeric CHGs offers promising advancements, yet several challenges must be addressed. High computational costs, particularly for deep learning models, hinder scalability and real‐time use in biomedical applications. Data availability and preprocessing issues, such as fragmented datasets, require techniques like data augmentation to ensure model reliability. Overfitting remains a concern, necessitating rigorous validation methods to improve generalization. Furthermore, regulatory barriers delay adoption, as ML‐based hydrogels require validation through clinical trials and standardized approval protocols. Addressing these challenges will facilitate more effective and ethical integration of ML in biomedical applications.

The future of AI‐assisted design and applications for graphene‐based CHGs is poised to advance significantly with the integration of emerging ML paradigms, such as Large Language Models (LLMs), Generative AI, and Agentic AI [249, 250]. LLMs, with their ability to process vast amounts of scientific literature and experimental data, can accelerate the discovery of novel hydrogel formulations by predicting optimal compositions and guiding experimental design [236]. By leveraging multimodal datasets, these models can refine the mechanical, electrical, and biocompatibility properties of graphene‐based hydrogels, thereby enabling researchers to develop more efficient and adaptive wearable sensors [251].

Generative AI, particularly deep generative models, can further enhance hydrogel engineering by designing new material structures with desired functionalities and optimizing performance for applications in rehabilitation, motion sensing, and human‐machine interfaces [252]. Such advancements will enable the autonomous generation of novel graphene hydrogel configurations tailored for specific biomedical applications, significantly reducing the time and cost associated with traditional trial‐and‐error approaches [253]. Beyond material discovery, the incorporation of Agentic AI, which consists of AI systems capable of autonomous decision‐making, can revolutionize real‐time adaptation and control in wearable hydrogel‐based devices [254]. These intelligent agents can dynamically adjust sensing parameters based on user activity, thereby improving the accuracy and responsiveness of gesture recognition and rehabilitation monitoring [255, 256]. Additionally, enhancing the interpretability of ML models is crucial for ensuring trust and reliability in AI‐assisted biomedical applications. Explainable AI (XAI) techniques can provide insights into model decisions, allowing researchers and clinicians to understand how AI interprets sensor signals and gesture classifications, fostering greater adoption in healthcare settings. By integrating these advanced AI frameworks, graphene‐based CHGs hydrogels can evolve into more adaptive, intelligent, and interpretable systems, paving the way for next‐generation bio‐interfacing technologies with broad applications in personalized medicine, neurorehabilitation, and intelligent prosthetics.

## 7 Challenges, Scalability, and Future Directions for Commercialization and Clinical Translation

Although graphene‐integrated polymeric CHGs have demonstrated strong potential in laboratory‐scale biomedical studies, moving these systems toward large‐scale pilot production and industrial use remains challenging [257]. Consistently dispersing graphene without agglomeration, identifying concentrations that enhance performance while preserving biocompatibility, ensuring long‐term stability under physiological conditions, and establishing reliable, scalable crosslinking methods are all critical hurdles that must be addressed to enable successful commercialization. For example, graphene's limited aqueous dispersibility often leads to non‐uniform distribution within hydrogel networks, which can compromise key properties such as tensile strength, critical for applications like tissue scaffolds and drug delivery systems. In addition, the high production costs associated with techniques such as chemical vapor deposition (CVD), along with the risk of introducing defects during substrate transfer, remain major barriers to scale‐up. Commercialization is further constrained by regulatory requirements related to nanomaterial safety, including concerns about toxicity, bioaccumulation, and environmental impact under standards such as ISO 10993 [258, 259, 260, 261].

Pilot‐ and industrial‐scale production can be improved by optimizing processing parameters. Hydrothermal treatments at about 180°C for 12 h in sealed autoclaves can generate self‐assembled 3D graphene hydrogels with tunable pore sizes, supporting better dispersion and structural integrity [262]. Large‐scale synthesis of rGO becomes safer and more efficient when heat release during oxidation is controlled, for example, by ensuring dissolution heat dominates in sulfuric acid–based processes, enabling production rates up to 2 kg/day with conductivities near 600 S/m and surface areas of 400–600 m^2^/g [263]. Performance can be further tuned by adjusting GO/GNP ratios in dispersions, while hydrophilic coatings such as polydopamine improve graphene compatibility with water‐based hydrogel systems. Emerging strategies, including AI‐guided process optimization and roll‐to‐roll manufacturing, may support cost‐effective production at scales approaching 100 kg/h, although minimizing structural defects will remain an essential requirement [263, 264].

Progress in assessing the safety and efficacy of graphene‐based polymeric hydrogels remains largely at the proof‐of‐concept and in vitro stages, with early preclinical animal studies beginning to emerge [260]. Rodent models, such as rat tibialis anterior muscle defects, have shown that conductive graphene hydrogels can enhance skeletal muscle regeneration by improving vascularization, limiting inflammation over 4–12 weeks, and supporting myoblast differentiation, although long‐term studies remain limited due to variability in graphene purity [265]. In drug delivery, mouse tumor models have demonstrated controlled DOX release from graphene–alginate composites with reduced tumor growth and no acute toxicity, while ex vivo porcine skin studies for biosensing applications highlight the need for further evaluation of biodistribution and systemic effects in larger animals [266]. Wound‐healing models using PVA–montmorillonite CHGs have also reported faster closure, reduced scarring, and improved tensile strength without in vitro cytotoxicity.

Despite these promising findings across bone regeneration, neural scaffolds, and soft‐tissue repair, comprehensive preclinical validation in multiple species is still lacking. Current projections suggest that 5–10 years of additional study will be required before meeting safety and performance standards needed for human trials, including ISO 10993 testing, pharmacokinetic profiling, and regulatory evaluations by FDA or EMA [267]. These preclinical models remain essential for assessing immune compatibility, long‐term integration, and overall biosafety. With continued interdisciplinary efforts, applications such as wound dressings or localized drug delivery systems may be among the first candidates to advance toward early‐phase human studies within the coming decade [45].

## 8 Conclusion

Graphene‐based CHGs have emerged as a versatile class of biomaterials that combine the structural advantages of hydrogels with the exceptional electrical, mechanical, and thermal properties of graphene derivatives. This integration has enabled significant advancements across biomedical fields, particularly in tissue engineering, drug delivery, biosensing, and wound healing. In drug delivery, graphene enhances loading capacity and enables controlled, stimuli‐responsive release, supporting more precise and less toxic therapeutic interventions. In tissue engineering, its mechanical reinforcement and electrical conductivity contribute to scaffold designs that better support cell adhesion, proliferation, and lineage‐specific differentiation. Likewise, the intrinsic PT responsiveness of graphene has expanded the potential of hydrogels for localized cancer therapy and multimodal treatment strategies. Collectively, these characteristics illustrate the substantial potential of graphene‐based CHGs to address a wide spectrum of biomedical challenges through carefully tailored material design.

Despite these advances, the path toward clinical translation remains challenging. Persistent uncertainties surrounding long‐term biocompatibility, cytotoxicity, degradation behavior, and batch‐to‐batch variability highlight the need for more standardized approaches to material characterization and biological assessment. Scalable manufacturing and reproducible fabrication techniques also remain central obstacles, alongside the current lack of large‐animal studies or clinical trials required to establish biosafety and therapeutic benefit.

Looking ahead, meaningful progress will rely on the coordinated integration of materials engineering, biomedical science, and computational design. The development of graphene derivatives with improved biodegradability and reduced toxicity, supported by DFT modeling, ML frameworks, and predictive simulations, may accelerate the rational design of next‐generation CHGs. Emerging hybrid systems that integrate graphene with other 2DMats provide new opportunities to more precisely tailor electrical, mechanical, and PT performance for targeted biomedical functions. Likewise, the convergence of advanced fabrication technologies, such as 3D/4D bioprinting with patient‐specific design, holds promise for personalized regenerative therapies.

In summary, graphene‐based CHGs hold substantial promise as next‐generation platforms capable of advancing a wide range of biomedical applications. Realizing this potential will require coordinated interdisciplinary efforts focused on innovation, safety standardization, and translational research pathways that bridge laboratory discovery with clinical implementation. Overall, the alignment of advances in materials development with rigorous safety evaluation and application‐focused engineering will ultimately shape the extent to which graphene‐based CHGs can transition from experimental systems to clinically meaningful technologies.

## Author Contributions

A.H. contributed to conceptualization, methodology, data curation, formal analysis, investigation, validation, visualization, and writing of the original draft. I.B., K.S., and S.K.H.N. contributed to software, validation, and writing of the original draft. S.A., U.Z., and K.C. contributed to software, data curation, and visualization. S.H.P. contributed to administration, supervision, writing, reviewing, and editing.

## Conflicts of Interest

The authors declare no conflicts of interest.

## Declaration of Generative AI and AI‐Assisted Technologies in the Writing Process

During the preparation of this work, the author(s) used ChatGPT and Grok for grammatical correction. After using these tools, the author(s) reviewed and edited the content as needed and take full responsibility for the final content of the publication.

## Data Availability

The authors have nothing to report.
